# Supplementary material for: Point mutations in topoisomerase I alter the mutation spectrum in E. coli and impact the emergence of drug resistance genotypes
Source: Nucleic Acids Res. 2019 Nov 28;48(2):761–9. doi: 10.1093/nar/gkz1100 (PMC6954433; doi:10.1093/nar/gkz1100)
Supplement: gkz1100_Supplemental_File [file gkz1100_supplemental_file.zip › 190825_SI_combined.pdf]

Supplementary Tables & Figures

Supplementary Table 1 - Mutations identified in isolates from chemostat experiments
Supplementary Table 2 - Whole-genome sequencing of strains used in this study
Supplementary Table 3 - Mutations identified in DOG/AZT drug resistance assays
Supplementary Table 4 - Mutations identified in MA lines
Supplementary Table 5 - Mutations identified in D-cycloserine drug resistance assays

Supplementary Fig. 1 - AZT drug resistance assay
Supplementary Fig. 2 - Doubling rate of *topA* mutants in minimal media

Supplementary Item 1 - Phylogenetic analysis of bacterial topoisomerase I

Supplementary Table 1

Clone isolated from evolution experiment #1 - Antonovsky et al. (Cell 2016)

	gene	position	mutation	type	annotation	description
1	*pyrH*	189036	Δ3 bp	deletion	coding (695-697/726 nt)	UMP kinase is an essential enzyme involved in the de novo biosynthesis of pyrimidine nucleotides.
2	*brnQ*	415058	Δ2 bp	deletion	coding (12-13/1320 nt)	branched-chain amino acid transport system 2 carrier protein; LIV-II transport system for Ile, Leu, and Val
3	*queA*	420643	Δ2 bp	deletion	coding (177-178/1071 nt)	S-adenosylmethionine:tRNA ribosyltransferase-isomerase
4	*ybaM*	485622	Δ1 bp	deletion	coding (106/162 nt)	DUF2496 family protein
5	*prs*	1257049	C→T	point mutation	A95T (GCG→ACG)	phosphoribosylpyrophosphate synthase
6	*adhE*	1293152	Δ1 bp	deletion	coding (426/2676 nt)	fused acetaldehyde-CoA dehydrogenase/iron-dependent alcohol dehydrogenase/pyruvate-formate lyase deactivase
7	*yciV*	1317625	Δ1 bp	deletion	coding (149/882 nt)	PHP domain protein
8	*yciQ*	1319060	Δ2 bp	deletion	coding (58-59/1896 nt)	enhancer of membrane protein expression; putative inner membrane protein
9	*topA*	1325408	G→C	point mutation	R35P (CGC→CCC)	DNA topoisomerase I, omega subunit
10	*ldhA*	1436742	(GGTGAA)1→2	insertion (duplication)	coding (359/990 nt)	fermentative D-lactate dehydrogenase, NAD-dependent
11	*ydbA*	1465325	Δ2 bp	deletion	eudogene (552-553/3497	pseudogene, autotransporter homolog; interrupted byIS2 and IS30
12	*ydgC*	1676131	Δ1 bp	deletion	coding (157/336 nt)	GlpM family inner membrane protein
13	*slyA*	1714758	Δ3 bp	deletion	coding (322-324/435 nt)	global transcriptional regulator
14	*mepH*	1729616	Δ1 bp	deletion	intergenic (+109/-19)	murein DD-endopeptidase, space-maker hydrolase/superoxide dismutase, Fe
15	*ppsR*	1781885	Δ1 bp	deletion	coding (184/834 nt)	bifunctional regulatory protein: PEP synthase kinase and PEP synthase pyrophosphorylase
16	*sdiA*	1990393	Δ1 bp	deletion	intergenic (-80/+150)	quorum-sensing transcriptional activator/putative ABC superfamily transporter ATP-bindingsubunit
17	*dcm*	2024694	Δ4 bp	deletion	oding (1102-1105/1419 nt	DNA cytosine methyltransferase
18	*gatY*	2170739	(T)5→4	insertion	intergenic (-56/+252)	D-tagatose 1,6-bisphosphate aldolase 2, catalytic subunit/fructose-bisphosphate aldolase class I
19	*rseB*	2701729	Δ1 bp	deletion	coding (385/957 nt)	anti-sigma E factor, binds RseA
20	*truB*	3305544	Δ1 bp	deletion	coding (593/945 nt)	tRNA pseudouridine synthase B: tRNA pseudouridine(55) synthase and putative tmRNA pseudouridine(342) synthase
21	*yhdZ*	3416558	Δ1 bp	deletion	intergenic (+5/+224)	putative amino-acid transporter subunit/5S ribosomal RNA of rrnD operon
22	*kefG*	3474602	Δ3 bp	deletion	intergenic (-82/-44)	potassium-efflux system ancillary protein for KefB, glutathione-regulated/putative transporter subunit of ABC superfamily: ATP-binding component
23	*malQ*	3542161	Δ1 bp	deletion	coding (1269/2085 nt)	4-alpha-glucanotransferase (amylomaltase)
24	*gadW*	3658029	Δ2 bp	deletion	intergenic (-51/+316)	transcriptional activator of gadA and gadBC; repressor of gadX/acid resistance regulon transcriptional activator; autoactivator
25	*xylA*	3724422	(A)5→4	insertion	intergenic (-297/-69)	D-xylose isomerase/D-xylose transporter subunit
26	*sbp*	4099055	Δ1 bp	deletion	coding (294/990 nt)	sulfate transporter subunit
27	*trmA*	4152131	Δ2 bp	deletion	oding (1067-1068/1101 nt	tRNA m(5)U54 methyltransferase, SAM-dependent; tmRNA m(5)U341 methyltransferase
28	*xylE*	4232046	Δ1 bp	deletion	coding (137/1476 nt)	D-xylose transporter
29	*hflC*	4393393	Δ2 bp	deletion	coding (277-278/1005 nt)	modulator for HflB protease specific for phage lambda cII repressor
30	*lptG*	4478273	TGTTAGCAAAGTGTG)1-	insertion (duplication)	intergenic (+56/+105)	lipopolysaccharide export ABC permease of the LptBFGC export complex/DUF853 family protein with NTPase fold
31	*nanC*	4529912	G→T	point mutation	intergenic (-594/-862)	N-acetylnuraminic acid outer membrane channel protein/tyrosine recombinase/inversion of on/off regulator of fimA
32	*yjiY*	4581219	Δ3 bp	deletion	intergenic (-123/-253)	putative transporter/methyl-accepting chemotaxis protein I, serine sensor receptor
33	*nadR*	4617380	Δ1 bp	deletion	coding (249/1233 nt)	trifunctional protein: nicotinamide mononucleotide adenylyltransferase, ribosylnicotinamide kinase, transcriptional repressor

Clone isolated from evolution experiment #3 - Antonovsky et al. (Cell 2016)

	gene	position	mutation	type	annotation	description
1	*thrA*	1543	G→A	point mutation	G403S (GGT→AGT)	bifunctional aspartokinase/homoserine dehydrogenase 1
2	*pdhR*	119152	(ATTCGCGTC)1→2	insertion (duplication)	coding (574/765 nt)	pyruvate dehydrogenase complex repressor; autorepressor
3	*pyrH*	189041	(TAATGGGTGAAA)1→2	insertion (duplication)	coding (700/726 nt)	uridylate kinase
4	*brnQ*	415065	(AAGAT)1→2	insertion (duplication)	coding (19/1320 nt)	branched-chain amino acid transport system 2 carrier protein; LIV-II transport system for Ile, Leu, and Val
5	*ycbZ*	1013537	(TTGGCA)1→2	insertion (duplication)	coding (219/1761 nt)	putative peptidase
6	*opgH, yceK, msyB, mdtG*	1106933	Δ3,772 bp	deletion	large deletion	multiple genes
7	*prs*	1257022	TGATTGGTACACGAGC)1	insertion (duplication)	coding (310/948 nt)	phosphoribosylpyrophosphate synthase
8	*topA*	1325806	C→T	point mutation	R168C (CGT→TGT)	DNA topoisomerase I, omega subunit
9	*mlc*	1662559	(AGAGAAA)1→2	insertion (duplication)	coding (263/1221 nt)	glucosamine anaerobic growth regulon transcriptional repressor; autorepressor
10	*fliF*	2007006	CGAACTGCGTCTGCGCC	insertion (duplication)	coding (297/1659 nt)	flagellar basal-body MS-ring and collar protein
11	*yejM*	2278953	(GATGCCGAGCG)1→2	insertion (duplication)	coding (1099/1761 nt)	essential inner membrane DUF3413 domain-containing protein; lipid A production and membrane permeability factor
12	*yfiH*	2727902	(C)8→9	insertion	coding (492/732 nt)	UPF0124 family protein
13	*ptsP*	2961766	Δ5 bp	deletion	coding (24-28/2247 nt)	fused PTS enzyme: PEP-protein phosphotransferase(enzyme I)/GAF domain containing protein
14	*tufA*	3464372	+A	insertion	coding (317/1185 nt)	translation elongation factor EF-Tu 1
15	*crp*	3479546	(TAA)1→2	insertion (duplication)	coding (68/633 nt)	cAMP-activated global transcription factor, mediator of catabolite repression
16	*xylR*	3728788	TTCGTCAGCTTGTCGCC	insertion (duplication)	coding (450/1179 nt)	xylose divergent operon transcriptional activator
17	*xylE*	4231705	G→T	point mutation	R160S (CGC→AGC)	D-xylose transporter
18	*malE*	4236389	(CACCTA)1→2	insertion (duplication)	intergenic (-42/-323)	maltose transporter subunit/fused maltose transport subunit, ATP-binding component of ABC superfamily/regulatory protein
19	*malK*	4237722	GAACGACGTGGTGTTG	insertion (duplication)	coding (1011/1116 nt)	fused maltose transport subunit, ATP-binding component of ABC superfamily/regulatory protein
20	*yjgM*	4469423	TCTTACGTACGCAAGC)	insertion (duplication)	intergenic (-69/-124)	putative acetyltransferase/DUF898 family inner membrane protein
21	*yjiY*	4581222	Δ3 bp	deletion	intergenic (-126/-250)	putative transporter/methyl-accepting chemotaxis protein I, serine sensor receptor
22	*nadR*	4618035	G→A	point mutation	A302T (GCC→ACC)	trifunctional protein: nicotinamide mononucleotide adenylyltransferase, ribosylnicotinamide kinase, transcriptional repressor

Supplementary Table 2

topA+ - BW25133 *ΔsohB:kan*

	gene	position	mutation	type	annotation	description
1	*sohB*	1323587	Δ1032 bp	deletion	*ΔsohB:kan*	S49 peptidase family protein
2	*purP*	3888770	A→G	point mutation	I400I (ATT→ATC)	adenine permease, high affinity; adenine:H+ symporter
3	*rrsA*	4029087	A→T	point mutation	noncoding (197/1542 nt)	16S ribosomal RNA of rrnA operon

R35P - BW25133 *ΔsohB:kan topA* (g104c)

	gene	position	mutation	type	annotation	description
1	*dtpD*	736629	Δ1 bp	deletion	coding (1384/1482 nt)	dipeptide and tripeptide permease D
2	*sohB*	1323587	Δ1032 bp	deletion	*ΔsohB:kan*	S49 peptidase family protein
3	*topA*	1325408	G→C	point mutation	R35P (CGC→CCC)	DNA topoisomerase I, omega subunit
4	*yniD*	1799492	Δ2 bp	deletion	coding (71-72/108 nt)	uncharacterized protein
5	*rrsA*	4029087	A→T	point mutation	noncoding (197/1542 nt)	16S ribosomal RNA of rrnA operon
6	*dcuS*	4340277	+A	insertion	coding (1203/1632 nt)	sensory histidine kinase in two-component regulatory system with DcuR, regulator of anaerobic fumarate respiration

R35P - BW25133 *ΔsohB:kan topA* (g104c) *gyrA*

	gene	position	mutation	type	annotation	description
1	*prmC*	1262376	Δ1 bp	deletion	coding (827/834 nt)	N5-glutamine methyltransferase, modifies releasefactors RF-1 and RF-2
2	*sohB*	1323587	Δ1032 bp	deletion	*ΔsohB:kan*	S49 peptidase family protein
3	*topA*	1325408	G→C	point mutation	R35P (CGC→CCC)	DNA topoisomerase I, omega subunit
4	*yniD*	1799492	Δ2 bp	deletion	coding (71-72/108 nt)	uncharacterized protein
5	*gyrA / ubiG*	2332947	Δ1 bp	deletion	intergenic (-48/-99)	DNA gyrase (type II topoisomerase), subunit A/bifunctional 3-demethylubiquinone-9 3-methyltransferase/ 2-octaprenyl-6-hydroxy phenol methylase
6	*rrsA*	4029087	A→T	point mutation	noncoding (197/1542 nt)	16S ribosomal RNA of rrnA operon
7	*trmA*	4152756	Δ2 bp	deletion	coding (442-443/1101 nt)	sensory histidine kinase in two-component regulatory system with DcuR, regulator of anaerobic fumarate respiration

R168C - BW25133 *ΔsohB:kan topA* (c502t)

	gene	position	mutation	type	annotation	description
1	*sohB*	1323587	Δ1032 bp	deletion	*ΔsohB:kan*	S49 peptidase family protein
2	*topA*	1325806	C→T	point mutation	R168C (CGT→TGT)	DNA topoisomerase I, omega subunit
3	*rrsA*	4029087	A→T	point mutation	noncoding (197/1542 nt)	16S ribosomal RNA of rrnA operon

mutS - BW25133 *ΔmutS:kan*

	gene	position	mutation	type	annotation	description
1	*tgt*	422156	C→T	point mutation	G188G (GGC→GGT)	tRNA-guanine transglycosylase
2	*cyoA*	446783	A→G	point mutation	V95A (GTA→GCA)	cytochrome o ubiquinol oxidase subunit II
3	*mcbA*	837365	C→T	point mutation	D50N (GAT→AAT)	colanic acid mucoidy stimulation protein
4	*narH*	1280254	A→G	point mutation	T399A (ACC→GCC)	nitrate reductase 1, beta (Fe-S) subunit
5	*pgsA*	1985889	G→A	point mutation	A137V (GCG→GTG)	phosphatidylglycerophosphate synthetase
6	*pdxB*	2430483	T→C	point mutation	H283R (CAC→CGC)	erythronate-4-phosphate dehydrogenase
7	*evgS*	2478102	G→A	point mutation	A84T (GCT→ACT)	hybrid sensory histidine kinase in two-component regulatory system with EvgA
8	*ypfJ / purC*	2590129	G→A	point mutation	intergenic (-33/+135)	putative neutral zinc metallopeptidase/phosphoribosylaminoimidazole-succinocarboxamide synthetase
9	*mutS*	2850451	Δ2541 bp	deletion	Δ*mutS*:kan	methyl-directed mismatch repair protein
10	*hybO*	3138689	T→C	point mutation	N311S (AAC→AGC)	hydrogenase 2, small subunit
11	*ygiF / ygiM*	3194462	G→A	point mutation	intergenic (-138/-104)	inorganic triphosphatase/SH3 domain protein
12	*yiaF / yiaG*	3712762	T→C	point mutation	intergenic (-358/-76)	barrier effect co-colonization resistance factor; DUF3053 family lipoprotein/HTH_CROC1 family putative transcriptional regulator
13	*yibN / gpmM*	3778516	G→A	point mutation	intergenic (-141/-104)	putative rhodanese-related sulfurtransferase/phosphoglycero mutase III, cofactor-independent

Supplementary Table 3 (*galK*)

sample	strain	seq. gene	CDS Position	CDS Codon Number	DS Position Within Codo	type	change	protein Effect
**1**	*topA+*	*galK*	50	17	2	Del	-A	Frame Shift
**2**	*topA+*	*galK*	109	37	1	SNP	G -> A	D -> N
**3**	*topA+*	*galK*	185	62	2	Del	-GT	Frame Shift
**4**	*topA+*	*galK*	186	62	3	Del	-T	Frame Shift
**5**	*topA+*	*galK*	292	98	1	SNP	G -> T	G -> C
**6**	*topA+*	*galK*	377	126	2	SNP	G -> T	G -> V
**7**	*topA+*	*galK*	382	128	1	SNP	A -> T	S -> C
**8**	*topA+*	*galK*	388	130	1	SNP	T -> G	S -> A
**9**	*topA+*	*galK*	402	134	3	Del	-A	Frame Shift
**10**	*topA+*	*galK*	402	134	3	Del	-A	Frame Shift
**11**	*topA+*	*galK*	402	134	3	Del	-A	Frame Shift
**12**	*topA+*	*galK*	407	136	2	SNP	C -> A	A -> E
**13**	*topA+*	*galK*	457	153	1	SNP	C -> T	Truncation
**14**	*topA+*	*galK*	475	159	1	SNP	C -> T	Truncation
**15**	*topA+*	*galK*	484	162	1	SNP	G -> C	E -> Q
**16**	*topA+*	*galK*	501	167	3	Del	-C	Frame Shift
**17**	*topA+*	*galK*	520	174	1	SNP	G -> T	D -> Y
**18**	*topA+*	*galK*	523	175	1	SNP	C -> T	Truncation
**19**	*topA+*	*galK*	647	216	2	SNP	G -> T	R -> L
**20**	*topA+*	*galK*	787	263	1	SNP	A -> T	Truncation
**21**	*topA+*	*galK*	874	292	1	SNP	G -> T	Truncation
**22**	*topA+*	*galK*	909	303	3	Del	-TGATTTCGAAATCA	Frame Shift
**23**	*topA+*	*galK*	1036	346	1	SNP	G -> T	Truncation
**24**	*topA+*	*galK*	1099	367	1	SNP	A -> T	Truncation

sample	strain	seq. gene	CDS Position	CDS Codon Number	DS Position Within Codo	type	change	protein Effect
**1**	R35P	*galK*	60	20	60	Del	-TCACACC	Frame Shift
**2**	R35P	*galK*	63	21	63	Del	-CACC	Deletion
**3**	R35P	*galK*	65	22	65	Del	-CC	Frame Shift
**4**	R35P	*galK*	65	22	65	Del	-CC	Frame Shift
**5**	R35P	*galK*	65	22	65	Del	-CC	Frame Shift
**6**	R35P	*galK*	65	22	65	Del	-CC	Frame Shift
**7**	R35P	*galK*	65	22	65	Del	-CC	Frame Shift
**8**	R35P	*galK*	66	22	66	Del	-C	Frame Shift
**9**	R35P	*galK*	66	22	66	Del	-C	Frame Shift
**10**	R35P	*galK*	66	22	66	Del	-C	Frame Shift
**11**	R35P	*galK*	66	22	66	Del	-C	Frame Shift
**12**	R35P	*galK*	66	22	66	Del	-C	Frame Shift
**13**	R35P	*galK*	67	23	67	Del	-A	Frame Shift
**14**	R35P	*galK*	67	23	67	Del	-A	Frame Shift
**15**	R35P	*galK*	67	23	67	Del	-A	Frame Shift
**16**	R35P	*galK*	67	23	67	Del	-A	Frame Shift
**17**	R35P	*galK*	69	23	69	Del	-T	Frame Shift
**18**	R35P	*galK*	91	31	91	Del	-TT	Frame Shift
**19**	R35P	*galK*	91	31	91	Del	-TT	Frame Shift
**20**	R35P	*galK*	91	31	91	Del	-TTG	L ->
**21**	R35P	*galK*	148	50	148	Del	-T	Frame Shift
**22**	R35P	*galK*	804	268	804	Del	-AC	Frame Shift
**23**	R35P	*galK*	803	268	803	Del	-TA	Frame Shift
**24**	R35P	*galK*	809	270	809	Del	-CTGA	Frame Shift

sample	strain	seq. gene	CDS Position	CDS Codon Number	DS Position Within Codo	type	change	protein Effect
**1**	R168C	*galK*	65	22	2	Del	-CC	Frame Shift
**2**	R168C	*galK*	90	30	3	Del	-TTT	NL -> K
**3**	R168C	*galK*	90	30	3	Del	-TTTG	Frame Shift
**4**	R168C	*galK*	92	31	2	Del	(T)3 -> (T)2	Truncation
**5**	R168C	*galK*	95	32	2	Ins	CTGGCCGCGTGAATTT[illegible]	Frame Shift
**6**	R168C	*galK*	97	33	1	Del	-GGTG	Frame Shift
**7**	R168C	*galK*	114	38	3	SNP	C -> A	Truncation
**8**	R168C	*galK*	152	51	2	Ins	+TC	Frame Shift
**9**	R168C	*galK*	193	65	1	Ins	+GTAAAGTT	Frame Shift
**10**	R168C	*galK*	412	138	1	SNP	G -> T	Truncation
**11**	R168C	*galK*	548	183	2	Del	-AA	Frame Shift
**12**	R168C	*galK*	808	270	1	Ins	+TCATATACTG	Frame Shift
**13**	R168C	*galK*	808	270	1	Ins	+TCATATACTG	Frame Shift
**14**	R168C	*galK*	808	270	1	Ins	+TCATATACTG	Frame Shift

**15**	R168C	*galK*	809	270	2	Ins	+TATACTGA	Truncation
**16**	R168C	*galK*	809	270	2	Ins	+TATACTGA	Truncation
**17**	R168C	*galK*	809	270	2	Del	-CTGAAA	TEN -> N
**18**	R168C	*galK*	904	302	1	Ins	+TGCCTCTATG	Frame Shift
**19**	R168C	*galK*	985	329	1	Ins	+TGGC	Frame Shift
**20**	R168C	*galK*	1012	338	1	SNP	G -> A	G -> S
**21**	R168C	*galK*	1012	338	1	SNP	G -> T	G -> C
**22**	R168C	*galK*	1081	361	1	Del	-GAAGCAAAAAC	Frame Shift
**23**	R168C	*galK*	1091	364	2	Ins	+TATGAAGCAAAAA	Truncation
**24**	R168C	*galK*	1112	371	2	Ins	+GAGACTTTTT	Truncation

Supplementary Table 3 (*tdk*)

sample	strain	seq. gene	CDS position	CDS codon	codon frame	type	change	protein Effect
**1**	*topA+*	*tdk*	41	14	2	SNP	G -> T	G -> V
**2**	*topA+*	*tdk*	79	27	1	SNP	C -> T	Truncation
**3**	*topA+*	*tdk*	154	52	1	SNP	C -> T	R -> C
**4**	*topA+*	*tdk*	176	59	2	SNP	C -> A	A -> E
**5**	*topA+*	*tdk*	176	59	2	SNP	C -> A	A -> E
**6**	*topA+*	*tdk*	211	71	1	SNP	G -> T	Truncation
**7**	*topA+*	*tdk*	235	79	1	SNP	C -> T	Truncation
**8**	*topA+*	*tdk*	257	86	2	SNP	T -> G	V -> G
**9**	*topA+*	*tdk*	262	88	1	SNP	G -> T	Truncation
**10**	*topA+*	*tdk*	266	89	2	SNP	G -> T	C -> F
**11**	*topA+*	*tdk*	276	92	3	Ins	(T)5 -> (T)6	Frame Shift
**12**	*topA+*	*tdk*	310	104	1	SNP	G -> T	V -> F
**13**	*topA+*	*tdk*	314	105	2	SNP	A -> T	D -> V
**14**	*topA+*	*tdk*	314	105	2	SNP	A -> T	D -> V
**15**	*topA+*	*tdk*	343	115	1	SNP	G -> T	G -> C
**16**	*topA+*	*tdk*	343	115	1	SNP	G -> T	G -> C
**17**	*topA+*	*tdk*	384	128	3	SNP	C -> A	S -> R
**18**	*topA+*	*tdk*	403	135	1	SNP	T -> C	S -> P
**19**	*topA+*	*tdk*	426	142	3	SNP	A -> C	K -> N
**20**	*topA+*	*tdk*	428	143	2	Ins	(A)5 -> (A)6	Frame Shift
**21**	*topA+*	*tdk*	431	144	2	SNP	T -> G	I -> S
**22**	*topA+*	*tdk*	513	171	3	Del	-AATTGGTG	Frame Shift
**23**	*topA+*	*tdk*	515	172	2	Del	-TTGGTGGTA	IGGN -> N
**24**	*topA+*	*tdk*	516	172	3	Del	-TGGTGGTAAT	Frame Shift

sample	strain	seq. gene	CDS position	CDS codon	codon frame	type	change	protein Effect
**1**	R35P	*tdk*	267	89	3	SNP	C -> A	Truncation
**2**	R35P	*tdk*	13	5	1	Del	-T	Frame Shift
**3**	R35P	*tdk*	17	6	2	Del	-TCTACT	FYY -> Y
**4**	R35P	*tdk*	26	9	2	Del	-CCGC	Truncation
**5**	R35P	*tdk*	72	24	3	Del	-CAA	YN -> Y
**6**	R35P	*tdk*	72	24	3	Del	-CAA	YN -> Y
**7**	R35P	*tdk*	74	25	2	Del	-A	Frame Shift
**8**	R35P	*tdk*	74	25	2	Del	-AT	Frame Shift
**9**	R35P	*tdk*	74	25	2	Del	-AT	Frame Shift
**10**	R35P	*tdk*	76	26	1	Del	-T	Frame Shift
**11**	R35P	*tdk*	77	26	2	Del	-ACCA	Frame Shift
**12**	R35P	*tdk*	101	34	2	Del	-T	Frame Shift
**13**	R35P	*tdk*	103	35	1	Del	-G	Frame Shift
**14**	R35P	*tdk*	105	35	3	Del	-ATA	VY -> V
**15**	R35P	*tdk*	105	35	3	Del	-ATA	VY -> V
**16**	R35P	*tdk*	118	40	1	Del	(A)3 -> (A)2	Frame Shift
**17**	R35P	*tdk*	118	40	1	Del	(A)3 -> (A)2	Frame Shift
**18**	R35P	*tdk*	118	40	1	Del	(A)3 -> (A)2	Frame Shift
**19**	R35P	*tdk*	122	41	2	Del	-A	Frame Shift
**20**	R35P	*tdk*	183	61	3	Del	-A	Frame Shift
**21**	R35P	*tdk*	226	76	1	Del	-CA	Frame Shift
**22**	R35P	*tdk*	287	96	2	Del	-AAG	QV -> L
**23**	R35P	*tdk*	77	26	2	Del	-ACCA	Frame Shift
**24**	R35P	*tdk*	115	39	1	Del	-G	Frame Shift

sample	strain	seq. gene	CDS position	CDS codon	codon frame	type	change	protein Effect
**1**	R168C	*tdk*	524	175	2	Del	-A	Frame Shift
**2**	R168C	*tdk*	524	175	2	Del	-A	Frame Shift
**3**	R168C	*tdk*	25	9	1	Ins	+ACTAT	Frame Shift

**4**	R168C	*tdk*	27	9	3	Del	-C	Frame Shift
**5**	R168C	*tdk*	29	10	2	Ins	+TCTACTATTCCG	A -> VYYSA
**6**	R168C	*tdk*	74	25	2	Ins	+CTTCATACA	N -> TSYN
**7**	R168C	*tdk*	81	27	3	Ins	+AATTACCA	Frame Shift
**8**	R168C	*tdk*	81	27	3	Ins	+AATTACCA	Frame Shift
**9**	R168C	*tdk*	82	28	1	Del	-G	Frame Shift
**10**	R168C	*tdk*	93	31	3	SNP	G -> A	M -> I
**11**	R168C	*tdk*	167	56	2	SNP	C -> A	Truncation
**12**	R168C	*tdk*	184	62	1	Ins	+ATTA	Frame Shift
**13**	R168C	*tdk*	214	72	1	SNP	A -> T	I -> F
**14**	R168C	*tdk*	219	73	3	Ins	+AGATTCG	Frame Shift
**15**	R168C	*tdk*	290	97	2	Del	-T	Frame Shift
**16**	R168C	*tdk*	293	98	2	Del	-AT	Truncation
**17**	R168C	*tdk*	296	99	2	Del	-AA	Frame Shift
**18**	R168C	*tdk*	296	99	2	Del	-AA	Frame Shift
**19**	R168C	*tdk*	314	105	2	SNP	A -> T	D -> V
**20**	R168C	*tdk*	381	127	3	Ins	+TTATTGG	Frame Shift
**21**	R168C	*tdk*	385	129	1	Del	-CAATACT	Frame Shift
**22**	R168C	*tdk*	83	28	2	Ins	+TTACCAGG	Frame Shift
**23**	R168C	*tdk*	259	87	1	SNP	G -> A	D -> N
**24**	R168C	*tdk*	526	176	1	Ins	+TGGTGGTAAT	Truncation

Supplementary Table 4

mutations identified in *topA+* MA lines

	sample	line	passages	position	gene	mutation	length	type
**1**	*topA+*	A	50	484210	*mscK*	A→T	1	point mutation
**2**	*topA+*	B	50	508111	*ybaT*	T→G	1	point mutation
**3**	*topA+*	G	50	1137209	*rne*	G→A	1	point mutation
**4**	*topA+*	K	50	1636686	*cspF / quuQ*	(A)6→7	1	deletion
**5**	*topA+*	B	50	1818320	*cho*	T→G	1	point mutation
**6**	*topA+*	B	50	2413571	*yfcF*	C→T	1	point mutation
**7**	*topA+*	G	50	2760866	*rnlB / yfjP*	(C)7→6	1	deletion
**8**	*topA+*	F	50	2909352	*barA*	G→A	1	point mutation
**9**	*topA+*	G	50	2925627	*fucO*	G→A	1	point mutation
**10**	*topA+*	F	50	3116255	*glcB*	(T)5→4	1	deletion
**11**	*topA+*	E	50	3868626	*dgoR / yidX*	G→A	1	point mutation
**12**	*topA+*	J	50	4188941	*nfi*	C→T	1	point mutation
**13**	*topA+*	E	50	4531599	*fimB / fimE*	T→C	1	point mutation

mutations identified in R35P MA lines

	sample	line	passages	position	gene	mutation	len	type
**1**	R35P	E	25	64363	*polB*	Δ1 bp	1	deletion
**2**	R35P	E	25	79643	*leuA*	Δ1 bp	1	deletion
**3**	R35P	L	25	80750	*leuL / leuO*	Δ1 bp	1	deletion
**4**	R35P	D	25	164642	*fhuA*	Δ1 bp	1	deletion
**5**	R35P	C	25	216566	*rcsF / metQ*	Δ1 bp	1	deletion
**6**	R35P	B	25	217502	*metI*	Δ3 bp	3	deletion
**7**	R35P	D	25	217502	*metI*	Δ4 bp	4	deletion
**8**	R35P	E	25	217502	*metI*	Δ4 bp	4	deletion
**9**	R35P	L	25	217502	*metI*	Δ4 bp	4	deletion
**10**	R35P	D	25	218283	*metN*	Δ3 bp	3	deletion
**11**	R35P	K	25	219141	*metN / gmhB*	Δ4 bp	4	deletion
**12**	R35P	L	25	219591	*gmhB*	Δ1 bp	1	deletion
**13**	R35P	B	25	219614	*gmhB*	Δ1 bp	1	deletion
**14**	R35P	F	25	219617	*gmhB*	Δ4 bp	4	deletion
**15**	R35P	A	25	219731	*gmhB*	Δ1 bp	1	deletion
**16**	R35P	H	25	219731	*gmhB*	Δ3 bp	3	deletion
**17**	R35P	E	25	298198	*paoA*	Δ1 bp	1	deletion
**18**	R35P	B	25	322559	*betA*	Δ4 bp	4	deletion
**19**	R35P	E	25	342427	*yahO*	Δ2 bp	2	deletion
**20**	R35P	B	25	394413	*yaiW*	Δ1 bp	1	deletion
**21**	R35P	A	25	451407	*tig*	Δ1 bp	1	deletion
**22**	R35P	B	25	471171	*ybaY*	Δ75 bp	75	deletion
**23**	R35P	F	25	477727	*acrB*	Δ1 bp	1	deletion
**24**	R35P	K	25	480184	*acrA*	Δ1 bp	1	deletion
**25**	R35P	A	25	482103	*mscK*	Δ1 bp	1	deletion
**26**	R35P	H	25	482504	*mscK*	Δ1 bp	1	deletion
**27**	R35P	L	25	486795	*ybaN / apt*	Δ1 bp	1	deletion
**28**	R35P	H	25	588901	*cusS*	Δ3 bp	3	deletion
**29**	R35P	K	25	598569	*pheP*	Δ1 bp	1	deletion
**30**	R35P	E	25	607384	*fepA*	Δ2 bp	2	deletion
**31**	R35P	L	25	615297	*fepC*	Δ2 bp	2	deletion
**32**	R35P	D	25	692612	*metT / asnB*	Δ1 bp	1	deletion
**33**	R35P	A	25	695290	*umpH*	Δ2 bp	2	deletion
**34**	R35P	B	25	697333	*nagA*	Δ3 bp	3	deletion
**35**	R35P	H	25	707277	*ybfE*	Δ1 bp	1	deletion
**36**	R35P	L	25	708740	*seqA*	Δ2 bp	2	deletion
**37**	R35P	H	25	748516	*ybgD / gltA*	Δ1 bp	1	deletion
**38**	R35P	L	25	789914	*modE*	Δ1 bp	1	deletion
**39**	R35P	K	25	791714	*modB*	C→A	1	point mutation
**40**	R35P	F	25	830766	*ybiB*	Δ1 bp	1	deletion
**41**	R35P	B	25	841590	*glnQ*	Δ1 bp	1	deletion
**42**	R35P	H	25	856680	*ybiW*	Δ2 bp	2	deletion
**43**	R35P	F	25	919922	*clpA*	Δ3 bp	3	deletion
**44**	R35P	F	25	955143	*aroA*	Δ1 bp	1	deletion
**45**	R35P	H	25	956626	*ycaL / cmk*	Δ2 bp	2	deletion
**46**	R35P	K	25	956648	*ycaL / cmk*	Δ2 bp	2	deletion
**47**	R35P	C	25	986869	*pepN*	Δ1 bp	1	deletion
**48**	R35P	J	25	1043126	*gfcD*	Δ1 bp	1	deletion
**49**	R35P	L	25	1071784	*putA*	Δ1 bp	1	deletion
**50**	R35P	C	25	1072329	*putA*	Δ1 bp	1	deletion

**51**	R35P	A	25	1074281	*putA*	Δ1 bp	1	deletion
**52**	R35P	E	25	1120211	*mdtH*	Δ1 bp	1	deletion
**53**	R35P	H	25	1140232	*yceQ*	Δ1 bp	1	deletion
**54**	R35P	B	25	1141358	*rluC / yceF*	Δ1 bp	1	deletion
**55**	R35P	C	25	1162659	*ndh*	C→T	1	point mutation
**56**	R35P	L	25	1185891	*phoP*	Δ1 bp	1	deletion
**57**	R35P	E	25	1221551	*minC / ycgJ*	Δ2 bp	2	deletion
**58**	R35P	H	25	1277054	*narG*	Δ1 bp	1	deletion
**59**	R35P	J	25	1293171	*adhE*	Δ1 bp	1	deletion
**60**	R35P	K	25	1293171	*adhE*	Δ1 bp	1	deletion
**61**	R35P	B	25	1293172	*adhE*	Δ2 bp	2	deletion
**62**	R35P	L	25	1294797	*ychE / oppA*	Δ1 bp	1	deletion
**73**	R35P	H	25	1346397	*sapF*	Δ1 bp	1	deletion
**74**	R35P	C	25	1348752	*sapC*	Δ4 bp	4	deletion
**75**	R35P	E	25	1354673	*puuA*	Δ2 bp	2	deletion
**76**	R35P	J	25	1354674	*puuA*	Δ1 bp	1	deletion
**77**	R35P	H	25	1378534	*ycjX*	T→A	1	point mutation
**78**	R35P	E	25	1478871	*hrpA*	Δ1 bp	1	deletion
**79**	R35P	C	25	1479080	*hrpA*	Δ1 bp	1	deletion
**80**	R35P	D	25	1524575	*ydcD*	Δ5 bp	5	deletion
**81**	R35P	J	25	1623268	*rspR*	A→C	1	point mutation
**82**	R35P	H	25	1657000	*ynfF*	G→T	1	point mutation
**83**	R35P	A	25	1669605	*pntB*	Δ2 bp	2	deletion
**84**	R35P	F	25	1671561	*pntA*	Δ3 bp	3	deletion
**85**	R35P	A	25	1699900	*ydgK*	Δ2 bp	2	deletion
**86**	R35P	K	25	1700971	*rsxB*	Δ1 bp	1	deletion
**87**	R35P	H	25	1738346	*mdtK*	Δ3 bp	3	deletion
**88**	R35P	F	25	1762130	*ydiJ*	Δ1 bp	1	deletion
**89**	R35P	D	25	1798118	*arpB*	Δ2 bp	2	deletion
**90**	R35P	J	25	1798118	*arpB*	Δ2 bp	2	deletion
**91**	R35P	H	25	1853496	*ydjK*	Δ1 bp	1	deletion
**92**	R35P	C	25	1883959	*fadD*	Δ4 bp	4	deletion
**93**	R35P	H	25	1905513	*yebQ*	Δ1 bp	1	deletion
**94**	R35P	A	25	1910280	*msrC*	Δ1 bp	1	deletion
**95**	R35P	E	25	1910280	*msrC*	Δ1 bp	1	deletion
**96**	R35P	L	25	1910280	*msrC*	Δ1 bp	1	deletion
**97**	R35P	D	25	1945596	*yecD*	Δ3 bp	3	deletion
**98**	R35P	C	25	2094024	*gnd*	Δ2 bp	2	deletion
**99**	R35P	D	25	2127040	*wzc*	Δ1 bp	1	deletion
**100**	R35P	F	25	2135925	*udk*	Δ3 bp	3	deletion
**101**	R35P	F	25	2140837	*alkA*	Δ1 bp	1	deletion
**102**	R35P	E	25	2170219	*gatY*	Δ1 bp	1	deletion
**103**	R35P	L	25	2170219	*gatY*	Δ1 bp	1	deletion
**104**	R35P	J	25	2222186	*mdtQ*	Δ3 bp	3	deletion
**105**	R35P	D	25	2242919	*yeiE*	Δ1 bp	1	deletion
**106**	R35P	K	25	2273134	*bcr*	Δ2 bp	2	deletion
**107**	R35P	A	25	2338256	*yfaL / nrdA*	+T	1	insertion
**108**	R35P	B	25	2394029	*nuoF*	Δ1 bp	1	deletion
**109**	R35P	F	25	2400354	*lrhA / alaA*	Δ4 bp	4	deletion
**110**	R35P	B	25	2400384	*lrhA / alaA*	Δ3 bp	3	deletion
**111**	R35P	K	25	2400384	*lrhA / alaA*	Δ5 bp	5	deletion
**112**	R35P	J	25	2408387	*pta*	Δ3 bp	3	deletion
**113**	R35P	F	25	2437801	*epmC*	Δ1 bp	1	deletion
**114**	R35P	D	25	2455319	*fadL*	Δ1 bp	1	deletion
**115**	R35P	D	25	2512564	*yfeD*	Δ2 bp	2	deletion
**116**	R35P	A	25	2526864	*cysK / ptsH*	Δ1 bp	1	deletion
**117**	R35P	A	25	2593735	*gcvR*	Δ1 bp	1	deletion
**118**	R35P	J	25	2711722	*yfiF*	Δ3 bp	3	deletion
**119**	R35P	K	25	2711722	*yfiF*	Δ3 bp	3	deletion
**120**	R35P	J	25	2739238	*rimM*	Δ2 bp	2	deletion
**121**	R35P	J	25	2739311	*rpsP*	Δ5 bp	5	deletion
**122**	R35P	C	25	2782264	*ygaQ / csiD*	Δ1 bp	1	deletion
**123**	R35P	E	25	2823704	*srlQ*	Δ2 bp	2	deletion
**124**	R35P	J	25	2846466	*hypD*	Δ1 bp	1	deletion
**125**	R35P	B	25	2869582	*cysD*	Δ2 bp	2	deletion
**126**	R35P	C	25	2895229	*yqcE / ygcE*	G→A	1	point mutation
**127**	R35P	J	25	2904014	*mazG*	Δ1 bp	1	deletion
**128**	R35P	H	25	2907061	*rlmD*	Δ1 bp	1	deletion
**129**	R35P	L	25	2961764	*ptsP*	Δ5 bp	5	deletion
**130**	R35P	J	25	2961766	*ptsP*	Δ5 bp	5	deletion

**131**	R35P	L	25	3076019	*loiP*	Δ1 bp	1	deletion
**132**	R35P	D	25	3079755	*yqgC*	Δ2 bp	2	deletion
**133**	R35P	H	25	3079757	*yqgC*	Δ1 bp	1	deletion
**134**	R35P	H	25	3161060	*ygiS*	Δ3 bp	3	deletion
**135**	R35P	L	25	3189987	*hldE*	Δ2 bp	2	deletion
**136**	R35P	E	25	3197511	*bacA / folB*	Δ1 bp	1	deletion
**137**	R35P	K	25	3255378	*tdcE*	Δ1 bp	1	deletion
**138**	R35P	B	25	3321087	*rlmE / yhbY*	Δ1 bp	1	deletion
**139**	R35P	B	25	3321946	*greA*	Δ1 bp	1	deletion
**140**	R35P	L	25	3334727	*kdsD*	Δ3 bp	3	deletion
**141**	R35P	E	25	3372154	*rplM / yhcM*	Δ1 bp	1	deletion
**142**	R35P	H	25	3373135	*yhcM*	Δ1 bp	1	deletion
**143**	R35P	B	25	3472059	*yheV*	Δ8 bp	8	deletion
**144**	R35P	L	25	3474595	*kefG / yheS*	Δ5 bp	5	deletion
**145**	R35P	A	25	3511887	*aroB / aroK*	Δ2 bp	2	deletion
**146**	R35P	B	25	3511887	*aroB / aroK*	Δ2 bp	2	deletion
**147**	R35P	H	25	3511889	*aroB / aroK*	Δ1 bp	1	deletion
**148**	R35P	E	25	3512778	*aroK / hofQ*	Δ1 bp	1	deletion
**149**	R35P	H	25	3516623	*mrcA*	Δ3 bp	3	deletion
**150**	R35P	A	25	3516651	*mrcA*	Δ3 bp	3	deletion
**151**	R35P	H	25	3522797	*hslR*	Δ3 bp	3	deletion
**152**	R35P	E	25	3600007	*zntA*	Δ1 bp	1	deletion
**153**	R35P	B	25	3600008	*zntA*	Δ1 bp	1	deletion
**154**	R35P	A	25	3605813	*yhhT*	Δ1 bp	1	deletion
**155**	R35P	C	25	3700329	*dppA*	Δ1 bp	1	deletion
**156**	R35P	L	25	3706505	*tag*	Δ1 bp	1	deletion
**157**	R35P	C	25	3730574	*bax / malS*	Δ2 bp	2	deletion
**158**	R35P	E	25	3798750	*waaP*	Δ1 bp	1	deletion
**159**	R35P	C	25	3805800	*yicR*	Δ2 bp	2	deletion
**160**	R35P	H	25	3805800	*yicR*	Δ2 bp	2	deletion
**161**	R35P	K	25	3808124	*slmA*	Δ1 bp	1	deletion
**162**	R35P	L	25	3811240	*dinD*	Δ3 bp	3	deletion
**163**	R35P	J	25	3857724	*yidP*	T→G	1	point mutation
**164**	R35P	B	25	3927212	*rbsA*	Δ1 bp	1	deletion
**165**	R35P	K	25	3986034	*cyaA*	Δ2 bp	2	deletion
**166**	R35P	L	25	3986036	*cyaA*	Δ5 bp	5	deletion
**167**	R35P	F	25	3986038	*cyaA*	Δ3 bp	3	deletion
**168**	R35P	K	25	4018957	*ubiD*	Δ1 bp	1	deletion
**169**	R35P	D	25	4027224	*trkH*	Δ2 bp	2	deletion
**170**	R35P	F	25	4144253	*argE*	Δ2 bp	2	deletion
**171**	R35P	A	25	4145856	*argC*	Δ4 bp	4	deletion
**172**	R35P	A	25	4153822	*btuB*	Δ1 bp	1	deletion
**173**	R35P	E	25	4153822	*btuB*	Δ3 bp	3	deletion
**174**	R35P	K	25	4153823	*btuB*	Δ1 bp	1	deletion
**175**	R35P	C	25	4383675	*queG*	Δ2 bp	2	deletion
**176**	R35P	A	25	4391655	*hflX*	Δ2 bp	2	deletion
**177**	R35P	L	25	4395336	*purA*	C→A	1	point mutation
**178**	R35P	E	25	4476032	*pepA / lptF*	Δ1 bp	1	deletion
**179**	R35P	H	25	4598054	*rimI*	Δ2 bp	2	deletion
**180**	R35P	K	25	4617378	*nadR*	Δ2 bp	2	deletion
**181**	R35P	L	25	4630737	*yjjY / yjtD*	Δ8 bp	8	deletion

mutations identified in R35P *gyrA* MA lines

	sample	line	passages	position	gene	mutation	len	type
**1**	R35P *gyrA*	H	25	216566	*rcsF / metQ*	Δ1 bp	1	deletion
**2**	R35P *gyrA*	I	25	219445	*gmhB*	Δ1 bp	1	deletion
**3**	R35P *gyrA*	H	25	219689	*gmhB*	Δ6 bp	6	deletion
**4**	R35P *gyrA*	B	25	381741	*tauB*	G→A	1	point mutation
**5**	R35P *gyrA*	K	25	445794	*cyoB*	Δ4 bp	4	deletion
**6**	R35P *gyrA*	K	25	481709	*acrR*	Δ3 bp	3	deletion
**7**	R35P *gyrA*	F	25	622149	*entE*	Δ1 bp	1	deletion
**8**	R35P *gyrA*	B	25	693892	*asnB*	Δ4 bp	4	deletion
**9**	R35P *gyrA*	A	25	707321	*ybfE*	Δ1 bp	1	deletion
**10**	R35P *gyrA*	D	25	910766	*ybjE / aqpZ*	Δ1 bp	1	deletion
**11**	R35P *gyrA*	A	25	914832	*macA*	Δ1 bp	1	deletion
**12**	R35P *gyrA*	J	25	956437	*ycaL*	Δ3 bp	3	deletion
**13**	R35P *gyrA*	E	25	956438	*ycaL*	Δ1 bp	1	deletion
**14**	R35P *gyrA*	A	25	956624	*ycaL / cmk*	Δ4 bp	4	deletion
**15**	R35P *gyrA*	E	25	991914	*ssuA*	Δ1 bp	1	deletion
**16**	R35P *gyrA*	E	25	1043799	*gfcC*	G→C	1	point mutation

**17**	R35P *gyrA*	A	25	1104337	*opgC*	Δ2 bp	2	deletion
**18**	R35P *gyrA*	I	25	1140230	*yceQ*	Δ4 bp	4	deletion
**19**	R35P *gyrA*	J	25	1140232	*yceQ*	Δ1 bp	1	deletion
**20**	R35P *gyrA*	A	25	1140235	*yceQ*	Δ2 bp	2	deletion
**21**	R35P *gyrA*	F	25	1190269	*rluE*	+TTTA	4	insertion
**22**	R35P *gyrA*	B	25	1285191	*rssA*	Δ2 bp	2	deletion
**23**	R35P *gyrA*	A	25	1293170	*adhE*	Δ4 bp	4	deletion
**24**	R35P *gyrA*	J	25	1293171	*adhE*	Δ1 bp	1	deletion
**25**	R35P *gyrA*	J	25	1310657	*yciG / trpA*	A→T	1	point mutation
**35**	R35P *gyrA*	F	25	1621955	*ydfG*	Δ2 bp	2	deletion
**36**	R35P *gyrA*	B	25	1671992	*pntA*	Δ1 bp	1	deletion
**37**	R35P *gyrA*	A	25	1697041	*add*	Δ2 bp	2	deletion
**38**	R35P *gyrA*	K	25	1994377	*fliZ*	Δ1 bp	1	deletion
**39**	R35P *gyrA*	H	25	2096781	*wbbL*	Δ1 bp	1	deletion
**40**	R35P *gyrA*	H	25	2134027	*asmA*	Δ1 bp	1	deletion
**41**	R35P *gyrA*	K	25	2138566	*yegE*	Δ1 bp	1	deletion
**42**	R35P *gyrA*	H	25	2478974	*evgS*	T→G	1	point mutation
**43**	R35P *gyrA*	J	25	2514277	*gltX / valU*	Δ2 bp	2	deletion
**44**	R35P *gyrA*	D	25	2527790	*ptsI*	Δ5 bp	5	deletion
**45**	R35P *gyrA*	F	25	2527822	*ptsI*	Δ1 bp	1	deletion
**46**	R35P *gyrA*	F	25	2637746	*rlmN / ndk*	Δ1 bp	1	deletion
**47**	R35P *gyrA*	H	25	2904014	*mazG*	Δ1 bp	1	deletion
**48**	R35P *gyrA*	D	25	2907061	*rlmD*	Δ1 bp	1	deletion
**49**	R35P *gyrA*	H	25	2919082	*queF*	Δ1 bp	1	deletion
**50**	R35P *gyrA*	J	25	2921092	*ygdH / sdaC*	Δ3 bp	3	deletion
**51**	R35P *gyrA*	I	25	2961065	*ptsP*	Δ2 bp	2	deletion
**52**	R35P *gyrA*	B	25	3076019	*loiP*	Δ1 bp	1	deletion
**53**	R35P *gyrA*	K	25	3078389	*speA*	Δ1 bp	1	deletion
**54**	R35P *gyrA*	B	25	3158596	*parC*	Δ3 bp	3	deletion
**55**	R35P *gyrA*	A	25	3164065	*qseC*	Δ1 bp	1	deletion
**56**	R35P *gyrA*	B	25	3199122	*ttdR*	Δ1 bp	1	deletion
**57**	R35P *gyrA*	I	25	3333560	*mlaF / yrbG*	Δ1 bp	1	deletion
**58**	R35P *gyrA*	I	25	3334729	*kdsD*	Δ1 bp	1	deletion
**59**	R35P *gyrA*	H	25	3395441	*csrD*	Δ1 bp	1	deletion
**60**	R35P *gyrA*	E	25	3472486	*kefB*	Δ1 bp	1	deletion
**61**	R35P *gyrA*	D	25	3512774	*aroK / hofQ*	Δ1 bp	1	deletion
**62**	R35P *gyrA*	B	25	3516624	*mrcA*	Δ2 bp	2	deletion
**63**	R35P *gyrA*	I	25	3573425	*yhhX*	Δ1 bp	1	deletion
**64**	R35P *gyrA*	D	25	3628378	*yhiM*	Δ1 bp	1	deletion
**65**	R35P *gyrA*	E	25	3986034	*cyaA*	Δ2 bp	2	deletion
**66**	R35P *gyrA*	A	25	3986036	*cyaA*	Δ5 bp	5	deletion
**67**	R35P *gyrA*	H	25	3986036	*cyaA*	Δ1 bp	1	deletion
**68**	R35P *gyrA*	J	25	4026770	*trkH*	Δ2 bp	2	deletion
**69**	R35P *gyrA*	I	25	4147294	*argH*	Δ1 bp	1	deletion
**70**	R35P *gyrA*	D	25	4150430	*sthA*	Δ1 bp	1	deletion
**71**	R35P *gyrA*	E	25	4275848	*acs*	Δ1 bp	1	deletion
**72**	R35P *gyrA*	D	25	4509097	*insB1 / yjhU*	Δ1 bp	1	deletion
**73**	R35P *gyrA*	D	25	4566411	*yjiV*	G→C	1	point mutation
**74**	R35P *gyrA*	B	25	4598054	*rimI*	Δ4 bp	4	deletion

mutations identified in R168C MA lines

	sample	line	passages	position	gene	mutation	len	type
**1**	R168C	C	50	14338	*dnaJ*	20 bp→20 bp	20	insertion
**2**	R168C	J	50	444167	*cyoB*	(T)5→9	4	deletion
**3**	R168C	K	50	447631	*ampG*	GCAGAGCAGGCAGGAA	21	insertion
**4**	R168C	J	50	550282	*cysS*	C→T	1	point mutation
**5**	R168C	C	50	823060	*ybhF*	Δ4 bp	4	deletion
**6**	R168C	L	50	907407	*hcr*	Δ1 bp	1	deletion
**7**	R168C	E	50	937479	*dmsA*	G→A	1	point mutation
**19**	R168C	F	50	1436751	*ldhA*	T→C	1	point mutation
**20**	R168C	G	50	1445599	*tynA*	C→T	1	point mutation
**21**	R168C	L	50	1740738	*valV*	(G)5→6	1	deletion
**22**	R168C	C	50	1754820	*sufD*	A→T	1	point mutation
**23**	R168C	A	50	1779154	*ppsA*	C→A	1	point mutation
**24**	R168C	L	50	1820040	*spy / astE*	(A)7→6	1	deletion
**25**	R168C	G	50	1878252	*yeaV*	A→C	1	point mutation
**26**	R168C	K	50	2169955	*gatY*	(T)6→5	1	deletion
**27**	R168C	K	50	2203820	*yehQ*	G→T	1	point mutation
**28**	R168C	A	50	2310121	*rcsB*	A→T	1	point mutation
**29**	R168C	J	50	2488146	*yfdX / ypdI*	T→C	1	point mutation

**30**	R168C	F	50	3045691	*ubiI* / *ubiH*	+TAAA	4	insertion
**31**	R168C	J	50	3217700	*ebgA*	(CGGCGACTA)2→3	1	deletion
**32**	R168C	F	50	3260502	*tdcA* / *tdcR*	C→T	1	point mutation
**33**	R168C	A	50	3650347	*[hdeD]*	Δ18 bp	18	deletion
**34**	R168C	C	50	3762390	*yibW*	20 bp→20 bp	20	insertion
**35**	R168C	K	50	3762391	*yibW*	19 bp→19 bp	19	insertion
**36**	R168C	D	50	3788411	*waaF*	Δ606 bp	606	deletion
**37**	R168C	H	50	3849301	*yidG*	Δ12 bp	12	deletion
**38**	R168C	A	50	3983793	*hemC*	(GTCATAGTT)1→2	9	insertion
**39**	R168C	F	50	4423107	*qorB*	Δ357 bp	357	deletion
**40**	R168C	G	50	4593622	*yjjQ*	C→T	1	point mutation

Supplementary Table 5

DCS resistance clones [R35P background]

	gene	position	mutation	type	annotation	description
clone #1	*bolA*	450138	Δ2 bp	deletion	coding (211-212/318 nt)	stationary-phase morphogene, transcriptional repressor for mreB; also regulator for dacA, dacC, and ampC
	ispB	3327579	+A / Δ1 bp	complex		octaprenyl diphosphate synthase

	gene	position	mutation	type	annotation	description
clone #2	*adhE*	1293172	Δ1 bp	deletion	coding (406/2676 nt)	fused acetaldehyde-CoA dehydrogenase/iron-dependent alcohol dehydrogenase/pyruvate-formate lyase deactivase
	ychE / oppA	1294755	Δ6 bp	deletion	intergenic (+54/-679)	UPF0056 family inner membrane protein/oligopeptide transporter subunit
	alaE	2792717	Δ2 bp	deletion	coding (195-196/450 nt)	alanine exporter, alanine-inducible, stress-responsive
	ispB	3327571	+GTT / Δ3 bp	complex		octaprenyl diphosphate synthase

	gene	position	mutation	type	annotation	description
clone #3	*ycgL*	1223216	Δ3 bp	deletion	coding (47-49/294 nt)	UPF0745 family protein
	lrhA / alaA	2400380	Δ2 bp	deletion	intergenic (-260/-659)	transcriptional repressor of flagellar, motility and chemotaxis genes/valine-pyruvate aminotransferase 2
	pta	2408387	Δ3 bp	deletion	coding (162-164/2145 nt)	phosphate acetyltransferase
	ispB	3327575	+A / Δ1 bp	complex		octaprenyl diphosphate synthase

	gene	position	mutation	type	annotation	description
clone #4	*lrp*	928051	Δ3 bp	deletion	coding (1-3/495 nt)	leucine-responsive global transcriptional regulator
	ychE / oppA	1295056	Δ3 bp	deletion	intergenic (+355/-381)	UPF0056 family inner membrane protein/oligopeptide transporter subunit
	yniD	1799493	Δ2 bp	deletion	coding (72-73/108 nt)	uncharacterized protein
	pgpC / yfhH	2692079	Δ2 bp	deletion	intergenic (-170/-38)	phosphatidylglycerophosphatase C, membrane bound/putative DNA-binding transcriptional regulator
	ispB	3327575	Δ3 + 3 bp	complex		octaprenyl diphosphate synthase
	nnr	4384090	Δ3 bp	deletion	coding (208-210/1548 nt)	bifunctional NAD(P)H-hydrate repair enzyme; C-terminal domain ADP-dependent (S)-NAD(P) H-hydrate dehydratase and N-terminal domain NAD(P)H-hydrate epimerase
	fecA	4504943	Δ1 bp	deletion	coding (1552/2325 nt)	ferric citrate outer membrane transporter

	gene	position	mutation	type	annotation	description
clone #5	*metN*	218246	Δ1 bp	deletion	coding (887/1032 nt)	DL-methionine transporter subunit
	ivy	236859	Δ1 bp	deletion	coding (30/474 nt)	inhibitor of c-type lysozyme, periplasmic
	nagK	1174694	Δ2 bp	deletion	coding (646-647/912 nt)	N-acetyl-D-glucosamine kinase
	ispB	3327575	Δ3 bp	deletion		octaprenyl diphosphate synthase

DCS resistance clones [mutS background]

	gene	position	mutation	type	annotation	description
clone #1	*allD*	541523	T→C	point mutation	K99K (AAA→AAG)	ureidoglycolate dehydrogenase
	ydcR	1505608	Δ1 bp	point mutation	coding (1349/1407 nt)	putative DNA-binding transcriptional regulator/putative amino transferase
	hisD	2085711	T→C	point mutation	C378C (TGT→TGC)	bifunctional histidinal dehydrogenase/ histidinol dehydrogenase
	ygjI	3220398	T→C	point mutation	V269A (GTA→GCA)	putative transporter
	yicC	3810482	T→C	point mutation	G149G (GGT→GGC)	UPF0701 family protein

	gene	position	mutation	type	annotation	description
clone #2	*lacI*	360752	A→G	point mutation	V331A (GTG→GCG)	DNA-binding transcriptional repressor
	ybiX	834375	C→T	point mutation	V97M (GTG→ATG)	Fe(II)-dependent oxygenase superfamily protein
	glnP	842181	C→T	point mutation	W132* (TGG→TAG)	glutamine transporter subunit
	mlrA	2208365	T→C	point mutation	G7G (GGT→GGC)	transcriptional activator of *csgD* and *csgBA*
	ascB	2835174	G→A	point mutation	A276T (GCT→ACT)	cryptic 6-phospho-beta-glucosidase
	ygfB	3048486	T→C	point mutation	A106A (GCA→GCG)	UPF0149 family protein
	mqsR	3161711	T→C	point mutation	T65A (ACT→GCT)	GCU-specific mRNA interferase toxin of the MqsR-MqsA toxin-antitoxin system; biofilm/motility regulator; anti-repressor
	gspH	3455199	G→A	point mutation	V125M (GTG→ATG)	putative general secretory pathway component, cryptic
	slyD	3471328	G→A	point mutation	H177Y (CAT→TAT)	FKBP-type peptidyl prolyl cis-trans isomerase (rotamase)
	yiaF / yiaG	3712762	T→C	point mutation	intergenic (-358/-76)	barrier effect co-colonization resistance factor; DUF3053 family lipoprotein/HTH_CROC1 family putative transcriptional regulator
	glmU	3907912	G→A	point mutation	P217S (CCG→TCG)	fused N-acetyl glucosamine-1-phosphate uridyltransferase/glucosamine-1-phosphate acetyl transferase

	hemN	4046560	G→A	point mutation	A386T (GCC→ACC)	coproporphyrinogen III oxidase, SAM and NAD(P)H dependent, oxygen-independent
	glnA	4050307	T→A	point mutation	D363V (GAT→GTT)	glutamine synthetase

	gene	position	mutation	type	annotation	description
clone #3	*dnaK*	12943	C→T	point mutation	R261C (CGC→TGC)	chaperone Hsp70, with co-chaperone DnaJ
	galM	784121	C→T	point mutation	S58N (AGC→AAC)	aldose 1-epimerase; type-1 mutarotase
	ybjS	901595	C→T	point mutation	M205I (ATG→ATA)	putative NAD(P)H-dependent oxidoreductase with NAD(P)-binding Rossmann-fold domain
	aat	922214	T→C	point mutation	V225V (GTA→GTG)	leucyl/phenylalanyl-tRNA-protein transferase
	rlmL	1003373	C→G	point mutation	A25G (GCC→GGC)	fused 23S rRNA m(2)G2445 and m(7)G2069 methyltransferase, SAM-dependent
	gfcB	1044421	C→T	point mutation	R123H (CGC→CAC)	O-antigen capsule production lipoprotein
	dauA	1256098	A→G	point mutation	S54S (AGT→AGC)	C4-dicarboxylic acid transporter
	ynjC	1832591	A→G	point mutation	S375G (AGC→GGC)	inner membrane putative ABC superfamily transporter permease
	uraA	2612267	A→G	point mutation	V418A (GTG→GCG)	uracil permease
	yphE	2670713	T→C	point mutation	K336K (AAA→AAG)	putative sugar transporter subunit of ABC superfamily, ATP-binding component
	barA	2908728	C→T	point mutation	P105S (CCC→TCC)	hybrid sensory histidine kinase, in two-component regulatory system with UvrY
	tas	2965763	A→G	point mutation	T270A (ACG→GCG)	putative NADP(H)-dependent aldo-keto reductase
	kefB	3473113	C→T	point mutation	G285E (GGG→GAG)	potassium:proton antiporter
	nirC	3491072	T→C	point mutation	G237G (GGT→GGC)	nitrite transporter
	yihO	4058129	C→T	point mutation	C333Y (TGT→TAT)	putative transporter
	murI	4155415	A→G	point mutation	P20P (CCA→CCG)	glutamate racemase
	fhuF	4594712	C→T	point mutation	G257S (GGC→AGC)	ferric iron reductase involved in ferric hydroximate transport

	gene	position	mutation	type	annotation	description
clone #4	*ybcF / purK*	546802	A→G	point mutation	intergenic (+14/+181)	putative carbonate kinase/N5-carboxyaminoimidazole ribonucleotide synthase
	sfmD	557552	A→G	point mutation	V800V (GTA→GTG)	putative outer membrane export usher protein
	sapB	1349556	C→T	point mutation	V58I (GTT→ATT)	antimicrobial peptide transport ABC transporter permease
	yeiE	2242834	G→A	point mutation	N88N (AAC→AAT)	putative DNA-binding transcriptional regulator
	pka	2713925	T→C	point mutation	L205P (CTG→CCG)	protein lysine acetyltransferase
	nanR	3367145	T→C	point mutation	D235G (GAC→GGC)	transcriptional repressor of the nan operon, induced by sialic acid

	gene	position	mutation	type	annotation	description
clone #5	*ftsA / ftsZ*	101774	A→G	point mutation	intergenic (+43/-18)	ATP-binding cell division protein involved in recruitment of FtsK to Z ring/GTP-binding tubulin-like cell division protein
	mlrA	2208365	T→C	point mutation	G7G (GGT→GGC)	transcriptional activator of *csgD* and *csgBA*
	hisS	2633757	T→C	point mutation	T60A (ACC→GCC)	histidyl tRNA synthetase
	yiaF / yiaG	3712762	T→C	point mutation	intergenic (-358/-76)	barrier effect co-colonization resistance factor; DUF3053 family lipoprotein/HTH_CROC1 family putative transcriptional regulator
	glnL	4049017	A→G	point mutation	L228P (CTA→CCA)	sensory histidine kinase in two-component regulatory system with GlnG
	yjcS	4294939	A→G	point mutation	G492G (GGT→GGC)	metallo-beta-lactamase superfamily protein
	fecE	4501099	A→G	point mutation	V59A (GTA→GCA)	iron-dicitrate transporter subunit

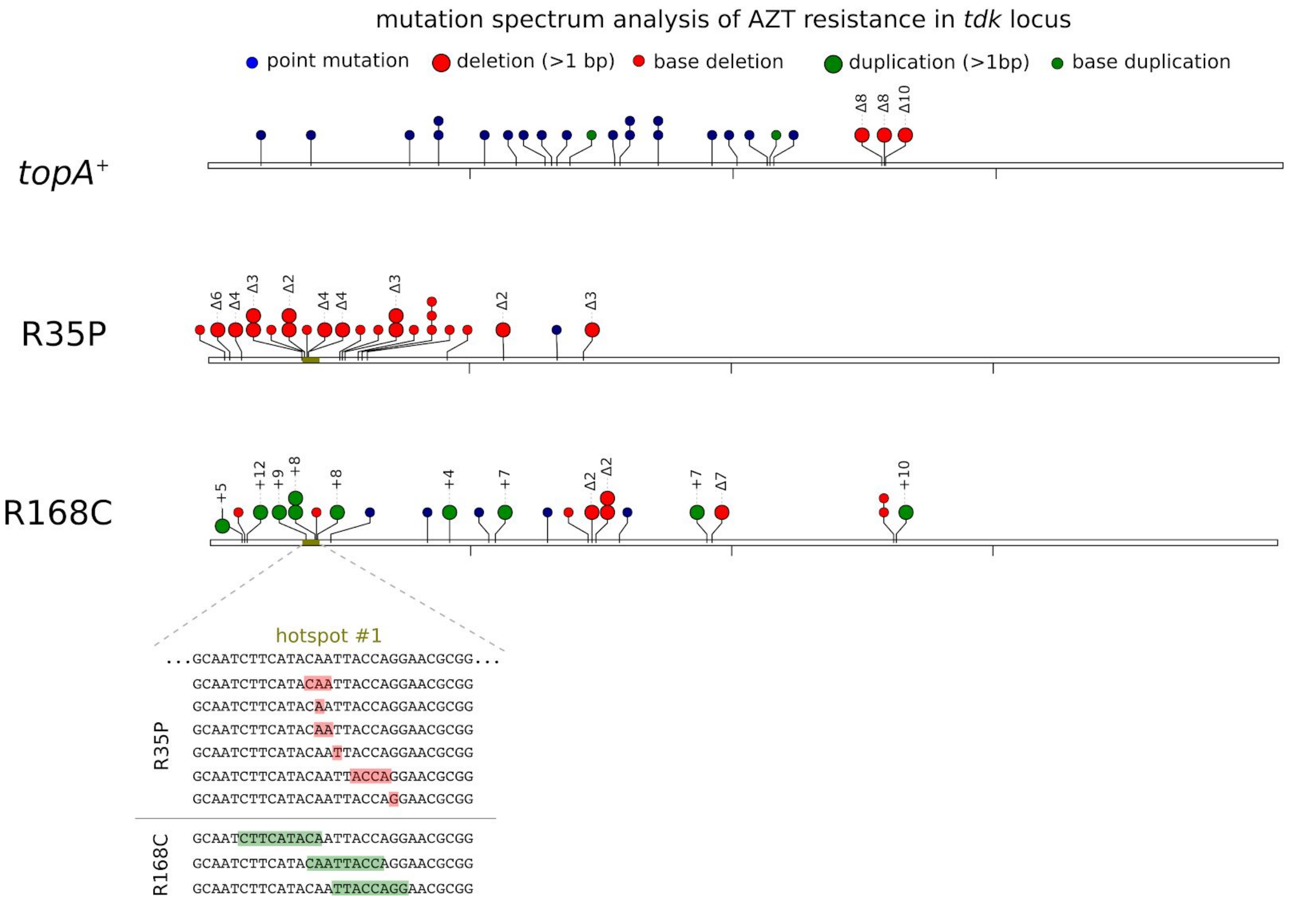

Supplementary figure 1. Drug resistance assay reveals an enrichment of insertion and deletion mutations in *topA* mutants. Mutation spectrum analysis of AZT resistant colonies arising in R168C and R35P mutant strains or from *topA*$^{+}$ strain. To determine the resistance conferring mutations, we used Sanger sequencing of PCR amplicons of the *tdk* locus. Overall, we sampled 24 independent resistant colonies for each genetic background. Notably, only a single colony was sampled from every assay to ensure that the analysis of independent mutational events.

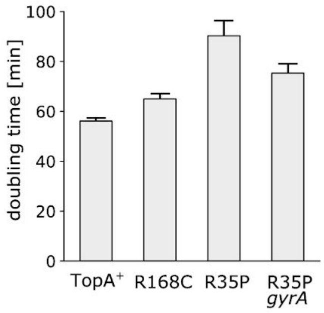

Supplementary figure 2. Doubling times of the *topA*+ stain in comparison to *topA* mutants in glucose supplemented M9 minimal media. Error bars represent standard deviation of biological repeats (n=6).

Supplementary Item 1

1750 sequences of bacterial topoisomerase I have been obtained from the EggNOG database (v.4.5.1, orthology group ENOG4105C73 using *E. coli* topoisomerase I as query). For each sequence we identify the residues at positions 35 and 168 based on the EggNOG precomputed alignment for this orthology group. Results were visualized using EvolView visualization tool (www.evolgenius.info) using the EggNOG precomputed tree for this orthology group. Green circles note ortholog sequences with R35/R168 residues. Red circles note sequences with other residues at these positions. Due to the size of the tree it is attached as a Supplementary Item file.
