# Supplementary material for: Point mutations in topoisomerase I alter the mutation spectrum in E. coli and impact the emergence of drug resistance genotypes
Source: Nucleic Acids Res. 2019 Nov 28;48(2):761–9. doi: 10.1093/nar/gkz1100 (PMC6954433; doi:10.1093/nar/gkz1100)
Supplement: gkz1100_Supplemental_File [file 190825_Sup_Item_1.pdf › 190825_Sup_Item_1.pdf]

Supplementary Item 1

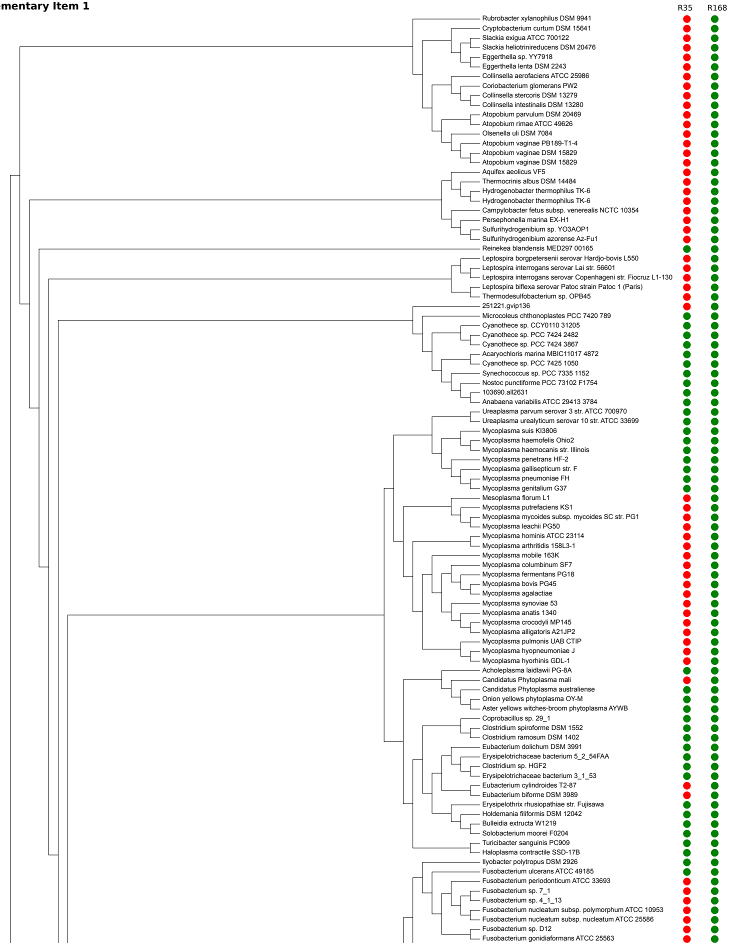

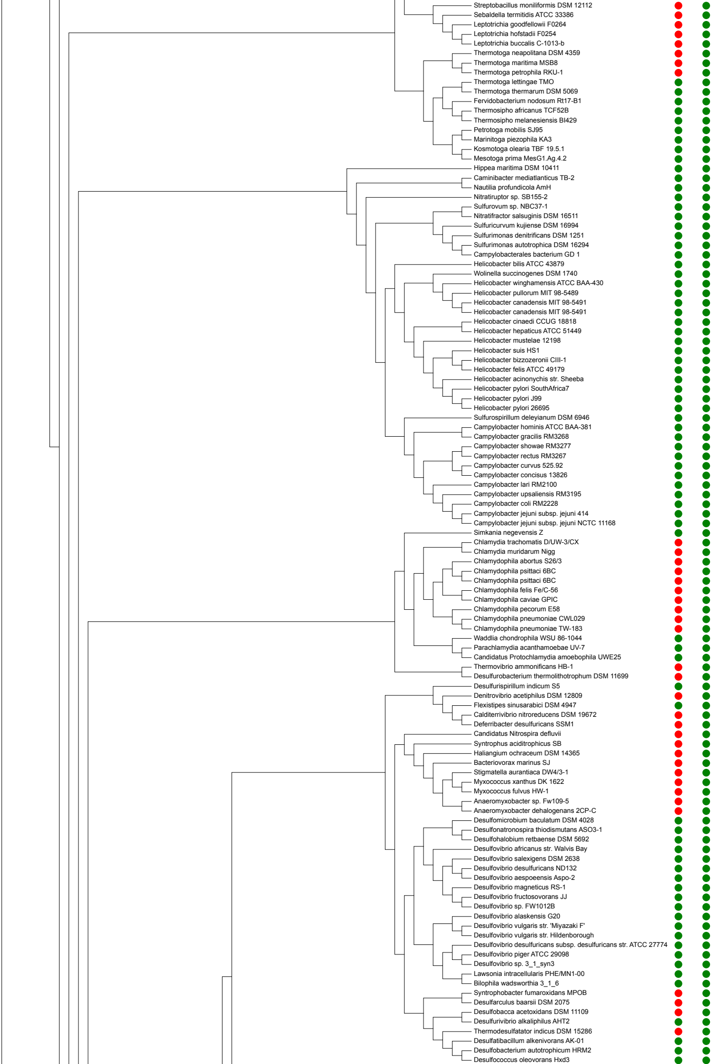
Streptobacillus moniliformis DSM 12112
Sebaldella termitidis ATCC 33386
Leptotrichia goodfellowii F0264
Leptotrichia hofstadii F0254
Leptotrichia buccalis C-1013-b
Thermotoga neapolitana DSM 4359
Thermotoga maritima MSB8
Thermotoga petrophila RKU-1
Thermotoga lettingae TMO
Thermotoga thermarum DSM 5069
Fervidobacterium nodosum Rt17-B1
Thermosipho africanus TCF52B
Thermosipho melanesiensis BI429
Petrotoga mobilis SJ95
Marinitoga piezophila KA3
Kosmotoga olearia TBF 19.5.1
Mesotoga prima MesG1.Ag.4.2
Hippea maritima DSM 10411
Caminibacter mediatlanticus TB-2
Nautilia profundicola AmH
Nitratiruptor sp. SB155-2
Sulfurovum sp. NBC37-1
Nitratifractor salsuginis DSM 16511
Sulfuricurvum kujiense DSM 16994
Sulfurimonas denitrificans DSM 1251
Sulfurimonas autotrophica DSM 16294
Campylobacterales bacterium GD 1
Helicobacter bilis ATCC 43879
Wolinella succinogenes DSM 1740
Helicobacter winghamensis ATCC BAA-430
Helicobacter pullorum MIT 98-5489
Helicobacter canadensis MIT 98-5491
Helicobacter canadensis MIT 98-5491
Helicobacter cinaedi CCUG 18818
Helicobacter hepaticus ATCC 51449
Helicobacter mustelae 12198
Helicobacter suis HS1
Helicobacter bizzozeronii CIII-1
Helicobacter felis ATCC 49179
Helicobacter acinonychis str. Sheeba
Helicobacter pylori SouthAfrica7
Helicobacter pylori J99
Helicobacter pylori 26695
Sulfurospirillum deleyianum DSM 6946
Campylobacter hominis ATCC BAA-381
Campylobacter gracilis RM3268
Campylobacter showae RM3277
Campylobacter rectus RM3267
Campylobacter curvus 525.92
Campylobacter concisus 13826
Campylobacter lari RM2100
Campylobacter upsaliensis RM3195
Campylobacter coli RM2228
Campylobacter jejuni subsp. jejuni 414
Campylobacter jejuni subsp. jejuni NCTC 11168
Simkania negevensis Z
Chlamydia trachomatis D/UW-3/CX
Chlamydia muridarum Nigg
Chlamydophila abortus S26/3
Chlamydophila psittaci 6BC
Chlamydophila psittaci 6BC
Chlamydophila felis Fe/C-56
Chlamydophila caviae GPIC
Chlamydophila pecorum E58
Chlamydophila pneumoniae CWL029
Chlamydophila pneumoniae TW-183
Waddlia chondrophila WSU 86-1044
Parachlamydia acanthamoebae UV-7
Candidatus Protochlamydia amoebophila UWE25
Thermovibrio ammonificans HB-1
Desulfurobacterium thermolithotrophum DSM 11699
Desulfurispirillum indicum S5
Denitrovibrio acetiphilus DSM 12809
Flexistipes sinusarabici DSM 4947
Calditerrivibrio nitroreducens DSM 19672
Deferribacter desulfuricans SSM1
Candidatus Nitrospira defluvii
Syntrophus aciditrophicus SB
Haliangium ochraceum DSM 14365
Bacteriovorax marinus SJ
Stigmatella aurantiaca DW4/3-1
Myxococcus xanthus DK 1622
Myxococcus fulvus HW-1
Anaeromyxobacter sp. Fw109-5
Anaeromyxobacter dehalogenans 2CP-C
Desulfomicrobium baculatum DSM 4028
Desulfonatronospira thiodismutans ASO3-1
Desulfohalobium retbaense DSM 5692
Desulfovibrio africanus str. Walvis Bay
Desulfovibrio salexigens DSM 2638
Desulfovibrio desulfuricans ND132
Desulfovibrio aespoeensis Aspo-2
Desulfovibrio magneticus RS-1
Desulfovibrio fructosovorans JJ
Desulfovibrio sp. FW1012B
Desulfovibrio alaskensis G20
Desulfovibrio vulgaris str. 'Miyazaki F'
Desulfovibrio vulgaris str. Hildenborough
Desulfovibrio desulfuricans subsp. desulfuricans str. ATCC 27774
Desulfovibrio piger ATCC 29098
Desulfovibrio sp. 3_1_syn3
Lawsonia intracellularis PHE/MN1-00
Bilophila wadsworthia 3_1_6
Syntrophobacter fumaroxidans MPOB
Desulfarculus baarsii DSM 2075
Desulfobacca acetoxidans DSM 11109
Desulfurivibrio alkaliphilus AHT2
Thermodesulfatator indicus DSM 15286
Desulfatibacillum alkenivorans AK-01
Desulfobacterium autotrophicum HRM2
Desulfococcus oleovorans Hxd3

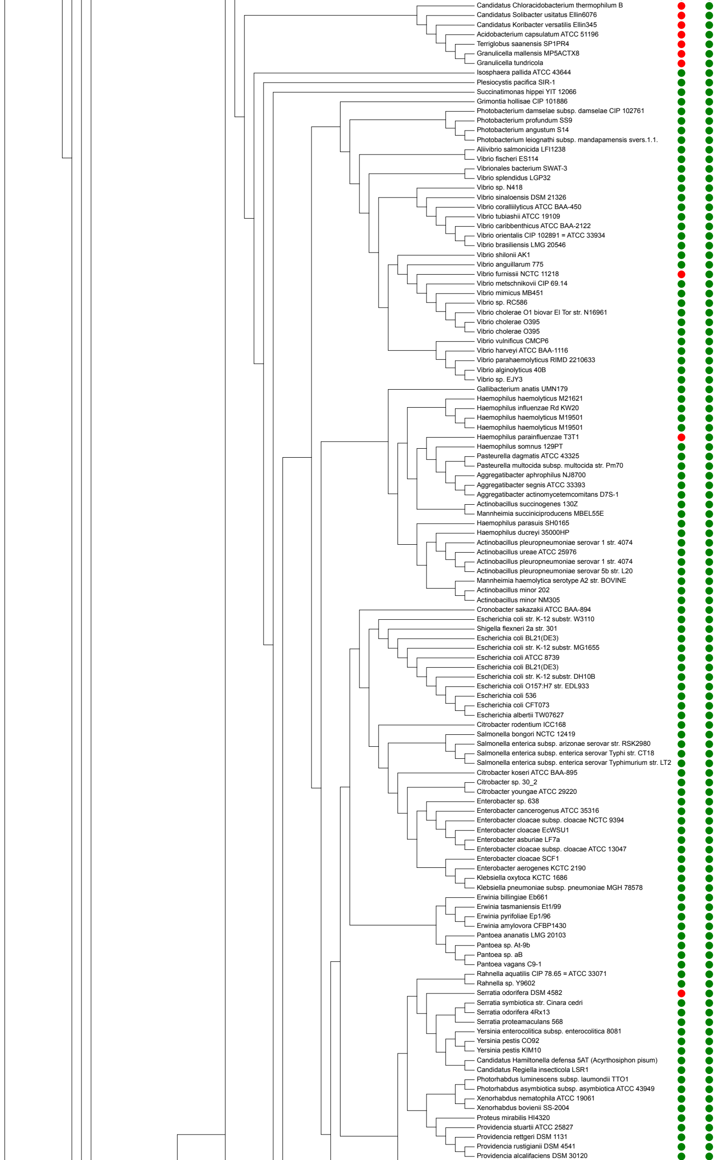
Candidatus Chloracidobacterium thermophilum B
Candidatus Solibacter usitatus Ellin6076
Candidatus Koribacter versatilis Ellin345
Acidobacterium capsulatum ATCC 51196
Terriglobus saanensis SP1PR4
Granulicella mallensis MP5ACTX8
Granulicella tundricola
Isosphaera pallida ATCC 43644
Plesiocystis pacifica SIR-1
Succinatimonas hippei YIT 12066
Grimontia hollisae CIP 101886
Photobacterium damselae subsp. damselae CIP 102761
Photobacterium profundum SS9
Photobacterium angustum S14
Photobacterium leiognathi subsp. mandapamensis svers.1.1.
Aliivibrio salmonicida LFI1238
Vibrio fischeri ES114
Vibrionales bacterium SWAT-3
Vibrio splendidus LGP32
Vibrio sp. N418
Vibrio sinaloensis DSM 21326
Vibrio coralliilyticus ATCC BAA-450
Vibrio tubiashii ATCC 19109
Vibrio caribbenthicus ATCC BAA-2122
Vibrio orientalis CIP 102891 = ATCC 33934
Vibrio brasiliensis LMG 20546
Vibrio shilonii AK1
Vibrio anguillarum 775
Vibrio furnissii NCTC 11218
Vibrio metschnikovii CIP 69.14
Vibrio mimicus MB451
Vibrio sp. RC586
Vibrio cholerae O1 biovar El Tor str. N16961
Vibrio cholerae O395
Vibrio cholerae O395
Vibrio vulnificus CMCP6
Vibrio harveyi ATCC BAA-1116
Vibrio parahaemolyticus RIMD 2210633
Vibrio alginolyticus 40B
Vibrio sp. EJY3
Gallibacterium anatis UMN179
Haemophilus haemolyticus M21621
Haemophilus influenzae Rd KW20
Haemophilus haemolyticus M19501
Haemophilus haemolyticus M19501
Haemophilus parainfluenzae T3T1
Haemophilus somnus 129PT
Pasteurella dagmatis ATCC 43325
Pasteurella multocida subsp. multocida str. Pm70
Aggregatibacter aphrophilus NJ8700
Aggregatibacter segnis ATCC 33393
Aggregatibacter actinomycetemcomitans D7S-1
Actinobacillus succinogenes 130Z
Mannheimia succiniciproducens MBEL55E
Haemophilus parasuis SH0165
Haemophilus ducreyi 35000HP
Actinobacillus pleuropneumoniae serovar 1 str. 4074
Actinobacillus ureae ATCC 25976
Actinobacillus pleuropneumoniae serovar 1 str. 4074
Actinobacillus pleuropneumoniae serovar 5b str. L20
Mannheimia haemolytica serotype A2 str. BOVINE
Actinobacillus minor 202
Actinobacillus minor NM305
Cronobacter sakazakii ATCC BAA-894
Escherichia coli str. K-12 substr. W3110
Shigella flexneri 2a str. 301
Escherichia coli BL21(DE3)
Escherichia coli str. K-12 substr. MG1655
Escherichia coli ATCC 8739
Escherichia coli BL21(DE3)
Escherichia coli str. K-12 substr. DH10B
Escherichia coli O157:H7 str. EDL933
Escherichia coli 536
Escherichia coli CFT073
Escherichia albertii TW07627
Citrobacter rodentium ICC168
Salmonella bongori NCTC 12419
Salmonella enterica subsp. arizonae serovar str. RSK2980
Salmonella enterica subsp. enterica serovar Typhi str. CT18
Salmonella enterica subsp. enterica serovar Typhimurium str. LT2
Citrobacter koseri ATCC BAA-895
Citrobacter sp. 30_2
Citrobacter youngae ATCC 29220
Enterobacter sp. 638
Enterobacter cancerogenus ATCC 35316
Enterobacter cloacae subsp. cloacae NCTC 9394
Enterobacter cloacae EcWSU1
Enterobacter asburiae LF7a
Enterobacter cloacae subsp. cloacae ATCC 13047
Enterobacter cloacae SCF1
Enterobacter aerogenes KCTC 2190
Klebsiella oxytoca KCTC 1686
Klebsiella pneumoniae subsp. pneumoniae MGH 78578
Erwinia billingiae Eb661
Erwinia tasmaniensis Et1/99
Erwinia pyrifoliae Ep1/96
Erwinia amylovora CFBP1430
Pantoea ananatis LMG 20103
Pantoea sp. At-9b
Pantoea sp. aB
Pantoea vagans C9-1
Rahnella aquatilis CIP 78.65 = ATCC 33071
Rahnella sp. Y9602
Serratia odorifera DSM 4582
Serratia symbiotica str. Cinara cedri
Serratia odorifera 4Rx13
Serratia proteamaculans 568
Yersinia enterocolitica subsp. enterocolitica 8081
Yersinia pestis CO92
Yersinia pestis KIM10
Candidatus Hamiltonella defensa 5AT (Acyrthosiphon pisum)
Candidatus Regiella insecticola LSR1
Photorhabdus luminescens subsp. laumondii TTO1
Photorhabdus asymbiotica subsp. asymbiotica ATCC 43949
Xenorhabdus nematophila ATCC 19061
Xenorhabdus bovienii SS-2004
Proteus mirabilis HI4320
Providencia stuartii ATCC 25827
Providencia rettgeri DSM 1131
Providencia rustigianii DSM 4541
Providencia alcalifaciens DSM 30120

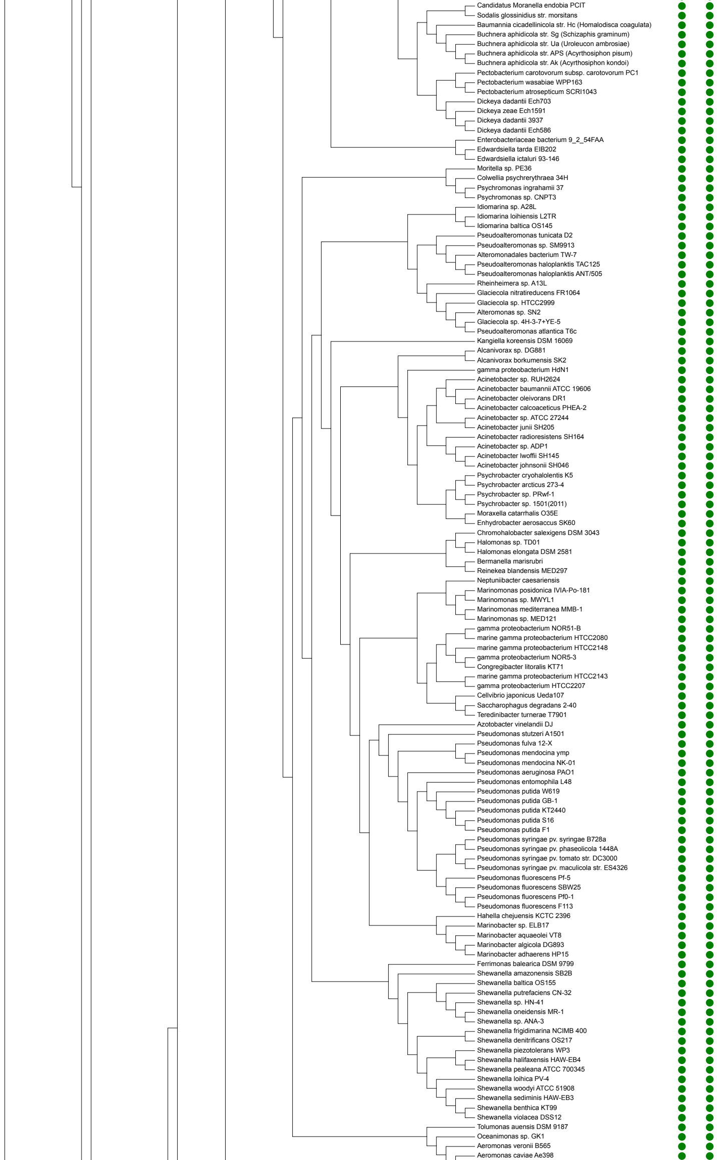

Candidatus Moranella endobia PCIT
Sodalis glossinidius str. morsitans
Baumannia cicadellinicola str. Hc (Homalodisca coagulata)
Buchnera aphidicola str. Sg (Schizaphis graminum)
Buchnera aphidicola str. Ua (Uroleucon ambrosiae)
Buchnera aphidicola str. APS (Acyrthosiphon pisum)
Buchnera aphidicola str. Ak (Acyrthosiphon kondoi)
Pectobacterium carotovorum subsp. carotovorum PC1
Pectobacterium wasabiae WPP163
Pectobacterium atrosepticum SCRI1043
Dickeya dadantii Ech703
Dickeya zeae Ech1591
Dickeya dadantii 3937
Dickeya dadantii Ech586
Enterobacteriaceae bacterium 9_2_54FAA
Edwardsiella tarda EIB202
Edwardsiella ictaluri 93-146
Moritella sp. PE36
Colwellia psychrerythraea 34H
Psychromonas ingrahamii 37
Psychromonas sp. CNPT3
Idiomarina sp. A28L
Idiomarina loihiensis L2TR
Idiomarina baltica OS145
Pseudoalteromonas tunicata D2
Pseudoalteromonas sp. SM9913
Alteromonadales bacterium TW-7
Pseudoalteromonas haloplanktis TAC125
Pseudoalteromonas haloplanktis ANT/505
Rheinheimera sp. A13L
Glaciecola nitratireducens FR1064
Glaciecola sp. HTCC2999
Alteromonas sp. SN2
Glaciecola sp. 4H-3-7+YE-5
Pseudoalteromonas atlantica T6c
Kangiella koreensis DSM 16069
Alcanivorax sp. DG881
Alcanivorax borkumensis SK2
gamma proteobacterium HdN1
Acinetobacter sp. RUH2624
Acinetobacter baumannii ATCC 19606
Acinetobacter oleivorans DR1
Acinetobacter calcoaceticus PHEA-2
Acinetobacter sp. ATCC 27244
Acinetobacter junii SH205
Acinetobacter radioresistens SH164
Acinetobacter sp. ADP1
Acinetobacter lwoffii SH145
Acinetobacter johnsonii SH046
Psychrobacter cryohalolentis K5
Psychrobacter arcticus 273-4
Psychrobacter sp. PRwf-1
Psychrobacter sp. 1501(2011)
Moraxella catarrhalis O35E
Enhydrobacter aerosaccus SK60
Chromohalobacter salexigens DSM 3043
Halomonas sp. TD01
Halomonas elongata DSM 2581
Bermanella marisrubri
Reinekea blandensis MED297
Neptuniibacter caesariensis
Marinomonas posidonica IVIA-Po-181
Marinomonas sp. MWYL1
Marinomonas mediterranea MMB-1
Marinomonas sp. MED121
gamma proteobacterium NOR51-B
marine gamma proteobacterium HTCC2080
marine gamma proteobacterium HTCC2148
gamma proteobacterium NOR5-3
Congregibacter litoralis KT71
marine gamma proteobacterium HTCC2143
gamma proteobacterium HTCC2207
Cellvibrio japonicus Ueda107
Saccharophagus degradans 2-40
Teredinibacter turnerae T7901
Azotobacter vinelandii DJ
Pseudomonas stutzeri A1501
Pseudomonas fulva 12-X
Pseudomonas mendocina ymp
Pseudomonas mendocina NK-01
Pseudomonas aeruginosa PAO1
Pseudomonas entomophila L48
Pseudomonas putida W619
Pseudomonas putida GB-1
Pseudomonas putida KT2440
Pseudomonas putida S16
Pseudomonas putida F1
Pseudomonas syringae pv. syringae B728a
Pseudomonas syringae pv. phaseolicola 1448A
Pseudomonas syringae pv. tomato str. DC3000
Pseudomonas syringae pv. maculicola str. ES4326
Pseudomonas fluorescens Pf-5
Pseudomonas fluorescens SBW25
Pseudomonas fluorescens Pf0-1
Pseudomonas fluorescens F113
Hahella chejuensis KCTC 2396
Marinobacter sp. ELB17
Marinobacter aquaeolei VT8
Marinobacter algicola DG893
Marinobacter adhaerens HP15
Ferrimonas balearica DSM 9799
Shewanella amazonensis SB2B
Shewanella baltica OS155
Shewanella putrefaciens CN-32
Shewanella sp. HN-41
Shewanella oneidensis MR-1
Shewanella sp. ANA-3
Shewanella frigidimarina NCIMB 400
Shewanella denitrificans OS217
Shewanella piezotolerans WP3
Shewanella halifaxensis HAW-EB4
Shewanella pealeana ATCC 700345
Shewanella loihica PV-4
Shewanella woodyi ATCC 51908
Shewanella sediminis HAW-EB3
Shewanella benthica KT99
Shewanella violacea DSS12
Tolumonas auensis DSM 9187
Oceanimonas sp. GK1
Aeromonas veronii B565
Aeromonas caviae Ae398

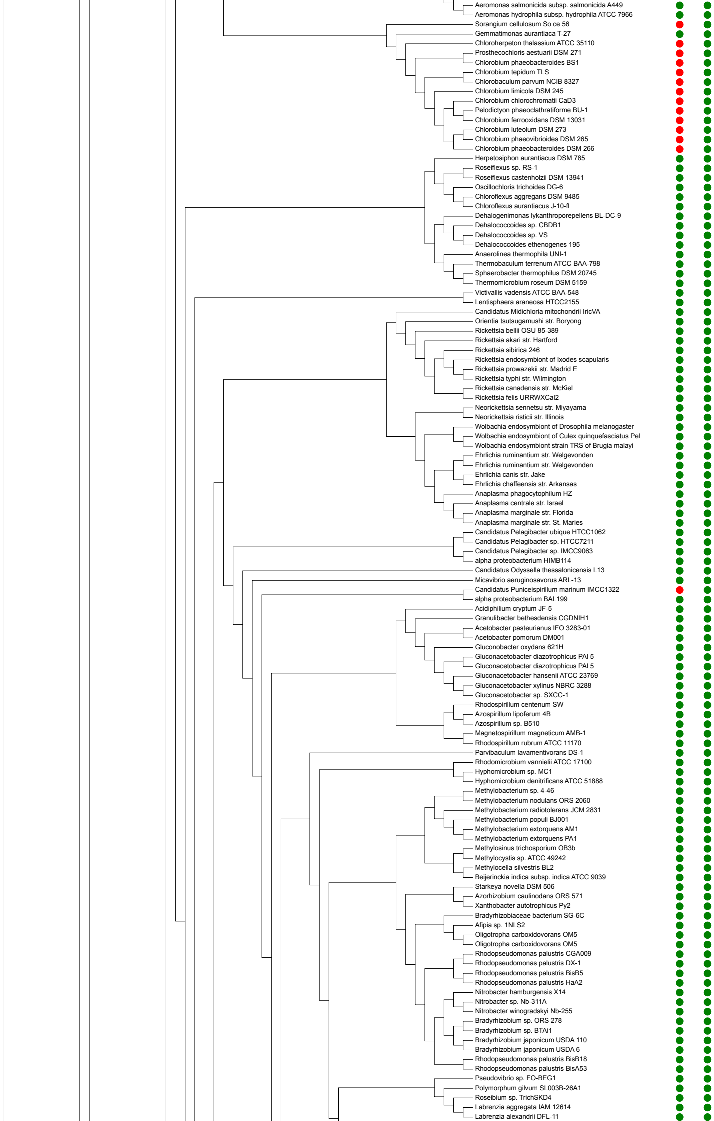
Aeromonas salmonicida subsp. salmonicida A449
Aeromonas hydrophila subsp. hydrophila ATCC 7966
Sorangium cellulosum So ce 56
Gemmatimonas aurantiaca T-27
Chloroherpeton thalassium ATCC 35110
Prosthecochloris aestuarii DSM 271
Chlorobium phaeobacteroides BS1
Chlorobium tepidum TLS
Chlorobaculum parvum NCIB 8327
Chlorobium limicola DSM 245
Chlorobium chlorochromatii CaD3
Pelodictyon phaeoclathratiforme BU-1
Chlorobium ferrooxidans DSM 13031
Chlorobium luteolum DSM 273
Chlorobium phaeovibrioides DSM 265
Chlorobium phaeobacteroides DSM 266
Herpetosiphon aurantiacus DSM 785
Roseiflexus sp. RS-1
Roseiflexus castenholzii DSM 13941
Oscillochloris trichoides DG-6
Chloroflexus aggregans DSM 9485
Chloroflexus aurantiacus J-10-fl
Dehalogenimonas lykanthroporepellens BL-DC-9
Dehalococcoides sp. CBDB1
Dehalococcoides sp. VS
Dehalococcoides ethenogenes 195
Anaerolinea thermophila UNI-1
Thermobaculum terrenum ATCC BAA-798
Sphaerobacter thermophilus DSM 20745
Thermomicrobium roseum DSM 5159
Victivallis vadensis ATCC BAA-548
Lentisphaera araneosa HTCC2155
Candidatus Midichloria mitochondrii IricVA
Orientia tsutsugamushi str. Boryong
Rickettsia bellii OSU 85-389
Rickettsia akari str. Hartford
Rickettsia sibirica 246
Rickettsia endosymbiont of Ixodes scapularis
Rickettsia prowazekii str. Madrid E
Rickettsia typhi str. Wilmington
Rickettsia canadensis str. McKiel
Rickettsia felis URRWXCal2
Neorickettsia sennetsu str. Miyayama
Neorickettsia risticii str. Illinois
Wolbachia endosymbiont of Drosophila melanogaster
Wolbachia endosymbiont of Culex quinquefasciatus Pel
Wolbachia endosymbiont strain TRS of Brugia malayi
Ehrlichia ruminantium str. Welgevonden
Ehrlichia ruminantium str. Welgevonden
Ehrlichia canis str. Jake
Ehrlichia chaffeensis str. Arkansas
Anaplasma phagocytophilum HZ
Anaplasma centrale str. Israel
Anaplasma marginale str. Florida
Anaplasma marginale str. St. Maries
Candidatus Pelagibacter ubique HTCC1062
Candidatus Pelagibacter sp. HTCC7211
Candidatus Pelagibacter sp. IMCC9063
alpha proteobacterium HIMB114
Candidatus Odyssella thessalonicensis L13
Micavibrio aeruginosavorus ARL-13
Candidatus Puniceispirillum marinum IMCC1322
alpha proteobacterium BAL199
Acidiphilium cryptum JF-5
Granulibacter bethesdensis CGDNIH1
Acetobacter pasteurianus IFO 3283-01
Acetobacter pomorum DM001
Gluconobacter oxydans 621H
Gluconacetobacter diazotrophicus PAl 5
Gluconacetobacter diazotrophicus PAl 5
Gluconacetobacter hansenii ATCC 23769
Gluconacetobacter xylinus NBRC 3288
Gluconacetobacter sp. SXCC-1
Rhodospirillum centenum SW
Azospirillum lipoferum 4B
Azospirillum sp. B510
Magnetospirillum magneticum AMB-1
Rhodospirillum rubrum ATCC 11170
Parvibaculum lavamentivorans DS-1
Rhodomicrobium vannielii ATCC 17100
Hyphomicrobium sp. MC1
Hyphomicrobium denitrificans ATCC 51888
Methylobacterium sp. 4-46
Methylobacterium nodulans ORS 2060
Methylobacterium radiotolerans JCM 2831
Methylobacterium populi BJ001
Methylobacterium extorquens AM1
Methylobacterium extorquens PA1
Methylosinus trichosporium OB3b
Methylocystis sp. ATCC 49242
Methylocella silvestris BL2
Beijerinckia indica subsp. indica ATCC 9039
Starkeya novella DSM 506
Azorhizobium caulinodans ORS 571
Xanthobacter autotrophicus Py2
Bradyrhizobiaceae bacterium SG-6C
Afipia sp. 1NLS2
Oligotropha carboxidovorans OM5
Oligotropha carboxidovorans OM5
Rhodopseudomonas palustris CGA009
Rhodopseudomonas palustris DX-1
Rhodopseudomonas palustris BisB5
Rhodopseudomonas palustris HaA2
Nitrobacter hamburgensis X14
Nitrobacter sp. Nb-311A
Nitrobacter winogradskyi Nb-255
Bradyrhizobium sp. ORS 278
Bradyrhizobium sp. BTAi1
Bradyrhizobium japonicum USDA 110
Bradyrhizobium japonicum USDA 6
Rhodopseudomonas palustris BisB18
Rhodopseudomonas palustris BisA53
Pseudovibrio sp. FO-BEG1
Polymorphum gilvum SL003B-26A1
Roseibium sp. TrichSKD4
Labrenzia aggregata IAM 12614
Labrenzia alexandrii DFL-11

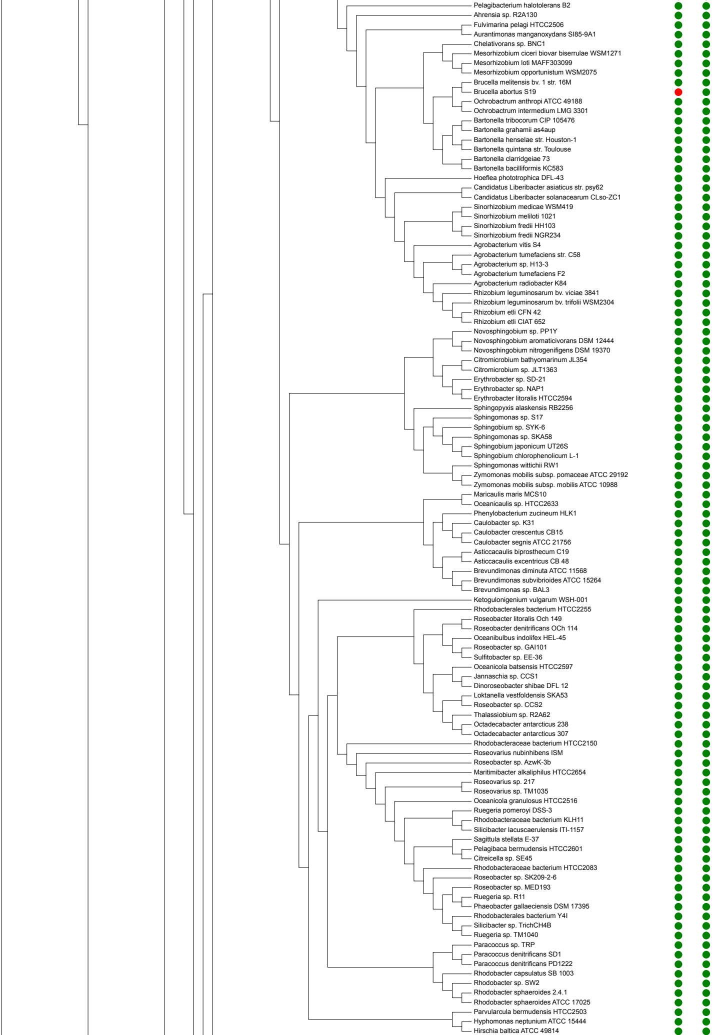
Pelagibacterium halotolerans B2
Ahrensia sp. R2A130
Fulvimarina pelagi HTCC2506
Aurantimonas manganoxydans SI85-9A1
Chelativorans sp. BNC1
Mesorhizobium ciceri biovar biserrulae WSM1271
Mesorhizobium loti MAFF303099
Mesorhizobium opportunistum WSM2075
Brucella melitensis bv. 1 str. 16M
Brucella abortus S19
Ochrobactrum anthropi ATCC 49188
Ochrobactrum intermedium LMG 3301
Bartonella tribocorum CIP 105476
Bartonella grahamii as4aup
Bartonella henselae str. Houston-1
Bartonella quintana str. Toulouse
Bartonella clarridgeiae 73
Bartonella bacilliformis KC583
Hoeflea phototrophica DFL-43
Candidatus Liberibacter asiaticus str. psy62
Candidatus Liberibacter solanacearum CLso-ZC1
Sinorhizobium medicae WSM419
Sinorhizobium meliloti 1021
Sinorhizobium fredii HH103
Sinorhizobium fredii NGR234
Agrobacterium vitis S4
Agrobacterium tumefaciens str. C58
Agrobacterium sp. H13-3
Agrobacterium tumefaciens F2
Agrobacterium radiobacter K84
Rhizobium leguminosarum bv. viciae 3841
Rhizobium leguminosarum bv. trifolii WSM2304
Rhizobium etli CFN 42
Rhizobium etli CIAT 652
Novosphingobium sp. PP1Y
Novosphingobium aromaticivorans DSM 12444
Novosphingobium nitrogenifigens DSM 19370
Citromicrobium bathyomarinum JL354
Citromicrobium sp. JLT1363
Erythrobacter sp. SD-21
Erythrobacter sp. NAP1
Erythrobacter litoralis HTCC2594
Sphingopyxis alaskensis RB2256
Sphingomonas sp. S17
Sphingobium sp. SYK-6
Sphingomonas sp. SKA58
Sphingobium japonicum UT26S
Sphingobium chlorophenolicum L-1
Sphingomonas wittichii RW1
Zymomonas mobilis subsp. pomaceae ATCC 29192
Zymomonas mobilis subsp. mobilis ATCC 10988
Maricaulis maris MCS10
Oceanicaulis sp. HTCC2633
Phenylobacterium zucineum HLK1
Caulobacter sp. K31
Caulobacter crescentus CB15
Caulobacter segnis ATCC 21756
Asticcacaulis biprosthecum C19
Asticcacaulis excentricus CB 48
Brevundimonas diminuta ATCC 11568
Brevundimonas subvibrioides ATCC 15264
Brevundimonas sp. BAL3
Ketogulonigenium vulgarum WSH-001
Rhodobacterales bacterium HTCC2255
Roseobacter litoralis Och 149
Roseobacter denitrificans OCh 114
Oceanibulbus indolifex HEL-45
Roseobacter sp. GAI101
Sulfitobacter sp. EE-36
Oceanicola batsensis HTCC2597
Jannaschia sp. CCS1
Dinoroseobacter shibae DFL 12
Loktanella vestfoldensis SKA53
Roseobacter sp. CCS2
Thalassiobium sp. R2A62
Octadecabacter antarcticus 238
Octadecabacter antarcticus 307
Rhodobacteraceae bacterium HTCC2150
Roseovarius nubinhibens ISM
Roseobacter sp. AzwK-3b
Maritimibacter alkaliphilus HTCC2654
Roseovarius sp. 217
Roseovarius sp. TM1035
Oceanicola granulosus HTCC2516
Ruegeria pomeroyi DSS-3
Rhodobacteraceae bacterium KLH11
Silicibacter lacuscaerulensis ITI-1157
Sagittula stellata E-37
Pelagibaca bermudensis HTCC2601
Citreicella sp. SE45
Rhodobacteraceae bacterium HTCC2083
Roseobacter sp. SK209-2-6
Roseobacter sp. MED193
Ruegeria sp. R11
Phaeobacter gallaeciensis DSM 17395
Rhodobacterales bacterium Y4I
Silicibacter sp. TrichCH4B
Ruegeria sp. TM1040
Paracoccus sp. TRP
Paracoccus denitrificans SD1
Paracoccus denitrificans PD1222
Rhodobacter capsulatus SB 1003
Rhodobacter sp. SW2
Rhodobacter sphaeroides 2.4.1
Rhodobacter sphaeroides ATCC 17025
Parvularcula bermudensis HTCC2503
Hyphomonas neptunium ATCC 15444
Hirschia baltica ATCC 49814

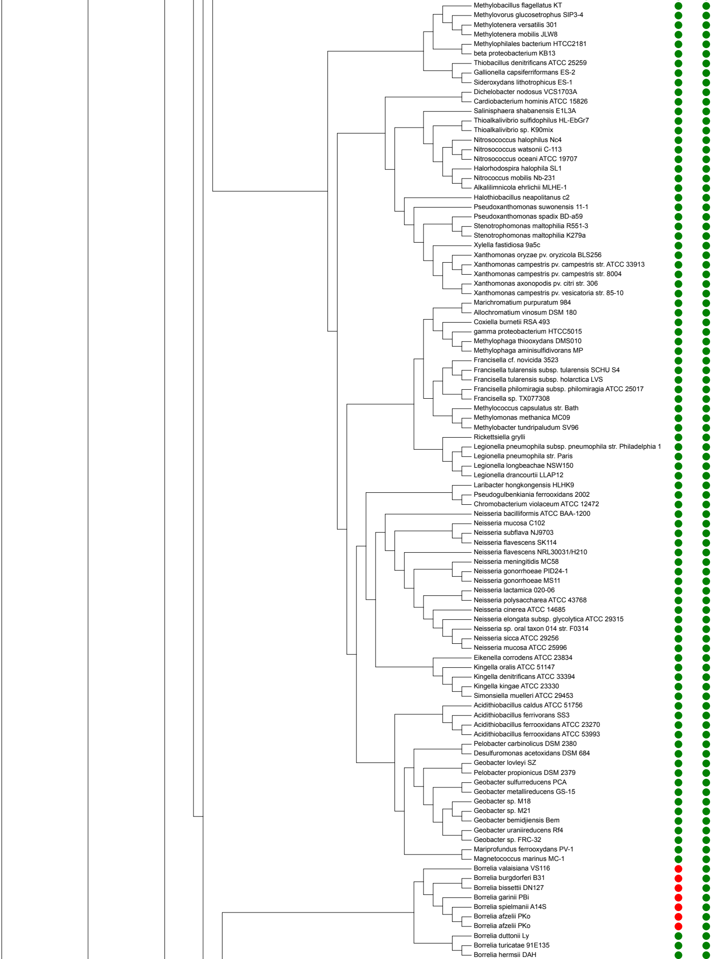
Methylobacillus flagellatus KT
Methylovorus glucosetrophus SIP3-4
Methylotenera versatilis 301
Methylotenera mobilis JLW8
Methylophilales bacterium HTCC2181
beta proteobacterium KB13
Thiobacillus denitrificans ATCC 25259
Gallionella capsiferriformans ES-2
Sideroxydans lithotrophicus ES-1
Dichelobacter nodosus VCS1703A
Cardiobacterium hominis ATCC 15826
Salinisphaera shabanensis E1L3A
Thioalkalivibrio sulfidophilus HL-EbGr7
Thioalkalivibrio sp. K90mix
Nitrosococcus halophilus Nc4
Nitrosococcus watsonii C-113
Nitrosococcus oceani ATCC 19707
Halorhodospira halophila SL1
Nitrococcus mobilis Nb-231
Alkalilimnicola ehrlichii MLHE-1
Halothiobacillus neapolitanus c2
Pseudoxanthomonas suwonensis 11-1
Pseudoxanthomonas spadix BD-a59
Stenotrophomonas maltophilia R551-3
Stenotrophomonas maltophilia K279a
Xylella fastidiosa 9a5c
Xanthomonas oryzae pv. oryzicola BLS256
Xanthomonas campestris pv. campestris str. ATCC 33913
Xanthomonas campestris pv. campestris str. 8004
Xanthomonas axonopodis pv. citri str. 306
Xanthomonas campestris pv. vesicatoria str. 85-10
Marichromatium purpuratum 984
Allochromatium vinosum DSM 180
Coxiella burnetii RSA 493
gamma proteobacterium HTCC5015
Methylophaga thiooxydans DMS010
Methylophaga aminisulfidivorans MP
Francisella cf. novicida 3523
Francisella tularensis subsp. tularensis SCHU S4
Francisella tularensis subsp. holarctica LVS
Francisella philomiragia subsp. philomiragia ATCC 25017
Francisella sp. TX077308
Methylococcus capsulatus str. Bath
Methylomonas methanica MC09
Methylobacter tundripaludum SV96
Rickettsiella grylli
Legionella pneumophila subsp. pneumophila str. Philadelphia 1
Legionella pneumophila str. Paris
Legionella longbeachae NSW150
Legionella drancourtii LLAP12
Laribacter hongkongensis HLHK9
Pseudogulbenkiania ferrooxidans 2002
Chromobacterium violaceum ATCC 12472
Neisseria bacilliformis ATCC BAA-1200
Neisseria mucosa C102
Neisseria subflava NJ9703
Neisseria flavescens SK114
Neisseria flavescens NRL30031/H210
Neisseria meningitidis MC58
Neisseria gonorrhoeae PID24-1
Neisseria gonorrhoeae MS11
Neisseria lactamica 020-06
Neisseria polysaccharea ATCC 43768
Neisseria cinerea ATCC 14685
Neisseria elongata subsp. glycolytica ATCC 29315
Neisseria sp. oral taxon 014 str. F0314
Neisseria sicca ATCC 29256
Neisseria mucosa ATCC 25996
Eikenella corrodens ATCC 23834
Kingella oralis ATCC 51147
Kingella denitrificans ATCC 33394
Kingella kingae ATCC 23330
Simonsiella muelleri ATCC 29453
Acidithiobacillus caldus ATCC 51756
Acidithiobacillus ferrivorans SS3
Acidithiobacillus ferrooxidans ATCC 23270
Acidithiobacillus ferrooxidans ATCC 53993
Pelobacter carbinolicus DSM 2380
Desulfuromonas acetoxidans DSM 684
Geobacter lovleyi SZ
Pelobacter propionicus DSM 2379
Geobacter sulfurreducens PCA
Geobacter metallireducens GS-15
Geobacter sp. M18
Geobacter sp. M21
Geobacter bemidjiensis Bem
Geobacter uraniireducens Rf4
Geobacter sp. FRC-32
Mariprofundus ferrooxydans PV-1
Magnetococcus marinus MC-1
Borrelia valaisiana VS116
Borrelia burgdorferi B31
Borrelia bissettii DN127
Borrelia garinii PBi
Borrelia spielmanii A14S
Borrelia afzelii PKo
Borrelia afzelii PKo
Borrelia duttonii Ly
Borrelia turicatae 91E135
Borrelia hermsii DAH

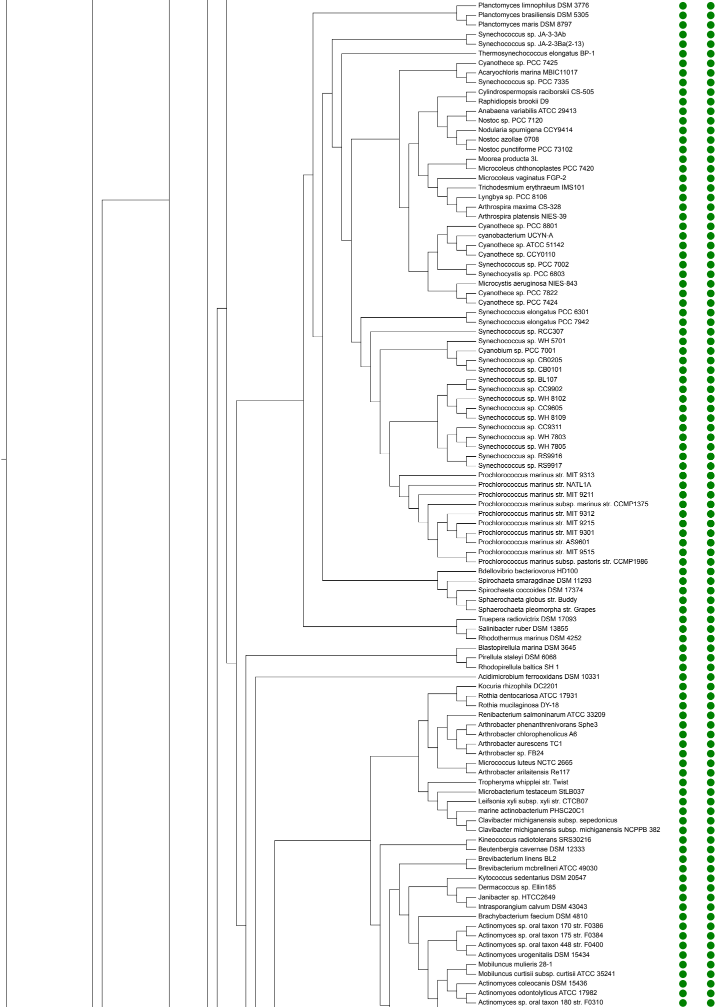

Planctomyces limnophilus DSM 3776
Planctomyces brasiliensis DSM 5305
Planctomyces maris DSM 8797
Synechococcus sp. JA-3-3Ab
Synechococcus sp. JA-2-3Ba(2-13)
Thermosynechococcus elongatus BP-1
Cyanothece sp. PCC 7425
Acaryochloris marina MBIC11017
Synechococcus sp. PCC 7335
Cylindrospermopsis raciborskii CS-505
Raphidiopsis brookii D9
Anabaena variabilis ATCC 29413
Nostoc sp. PCC 7120
Nodularia spumigena CCY9414
Nostoc azollae 0708
Nostoc punctiforme PCC 73102
Moorea producta 3L
Microcoleus chthonoplastes PCC 7420
Microcoleus vaginatus FGP-2
Trichodesmium erythraeum IMS101
Lyngbya sp. PCC 8106
Arthrospira maxima CS-328
Arthrospira platensis NIES-39
Cyanothece sp. PCC 8801
cyanobacterium UCYN-A
Cyanothece sp. ATCC 51142
Cyanothece sp. CCY0110
Synechococcus sp. PCC 7002
Synechocystis sp. PCC 6803
Microcystis aeruginosa NIES-843
Cyanothece sp. PCC 7822
Cyanothece sp. PCC 7424
Synechococcus elongatus PCC 6301
Synechococcus elongatus PCC 7942
Synechococcus sp. RCC307
Synechococcus sp. WH 5701
Cyanobium sp. PCC 7001
Synechococcus sp. CB0205
Synechococcus sp. CB0101
Synechococcus sp. BL107
Synechococcus sp. CC9902
Synechococcus sp. WH 8102
Synechococcus sp. CC9605
Synechococcus sp. WH 8109
Synechococcus sp. CC9311
Synechococcus sp. WH 7803
Synechococcus sp. WH 7805
Synechococcus sp. RS9916
Synechococcus sp. RS9917
Prochlorococcus marinus str. MIT 9313
Prochlorococcus marinus str. NATL1A
Prochlorococcus marinus str. MIT 9211
Prochlorococcus marinus subsp. marinus str. CCMP1375
Prochlorococcus marinus str. MIT 9312
Prochlorococcus marinus str. MIT 9215
Prochlorococcus marinus str. MIT 9301
Prochlorococcus marinus str. AS9601
Prochlorococcus marinus str. MIT 9515
Prochlorococcus marinus subsp. pastoris str. CCMP1986
Bdellovibrio bacteriovorus HD100
Spirochaeta smaragdinae DSM 11293
Spirochaeta coccoides DSM 17374
Sphaerochaeta globus str. Buddy
Sphaerochaeta pleomorpha str. Grapes
Truepera radiovictrix DSM 17093
Salinibacter ruber DSM 13855
Rhodothermus marinus DSM 4252
Blastopirellula marina DSM 3645
Pirellula staleyi DSM 6068
Rhodopirellula baltica SH 1
Acidimicrobium ferrooxidans DSM 10331
Kocuria rhizophila DC2201
Rothia dentocariosa ATCC 17931
Rothia mucilaginosa DY-18
Renibacterium salmoninarum ATCC 33209
Arthrobacter phenanthrenivorans Sphe3
Arthrobacter chlorophenolicus A6
Arthrobacter aurescens TC1
Arthrobacter sp. FB24
Micrococcus luteus NCTC 2665
Arthrobacter arilaitensis Re117
Tropheryma whipplei str. Twist
Microbacterium testaceum StLB037
Leifsonia xyli subsp. xyli str. CTCB07
marine actinobacterium PHSC20C1
Clavibacter michiganensis subsp. sepedonicus
Clavibacter michiganensis subsp. michiganensis NCPPB 382
Kineococcus radiotolerans SRS30216
Beutenbergia cavernae DSM 12333
Brevibacterium linens BL2
Brevibacterium mcbrellneri ATCC 49030
Kytococcus sedentarius DSM 20547
Dermacoccus sp. Ellin185
Janibacter sp. HTCC2649
Intrasporangium calvum DSM 43043
Brachybacterium faecium DSM 4810
Actinomyces sp. oral taxon 170 str. F0386
Actinomyces sp. oral taxon 175 str. F0384
Actinomyces sp. oral taxon 448 str. F0400
Actinomyces urogenitalis DSM 15434
Mobiluncus mulieris 28-1
Mobiluncus curtisii subsp. curtisii ATCC 35241
Actinomyces coleocanis DSM 15436
Actinomyces odontolyticus ATCC 17982
Actinomyces sp. oral taxon 180 str. F0310

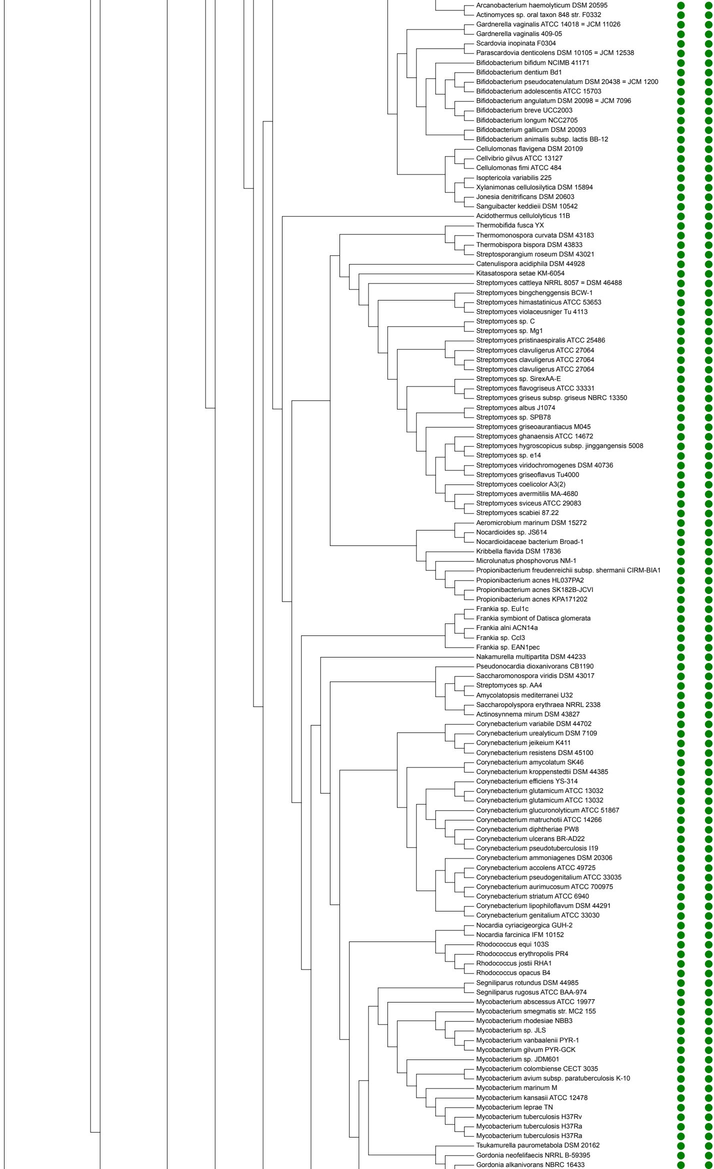
Arcanobacterium haemolyticum DSM 20595
Actinomyces sp. oral taxon 848 str. F0332
Gardnerella vaginalis ATCC 14018 = JCM 11026
Gardnerella vaginalis 409-05
Scardovia inopinata F0304
Parascardovia denticolens DSM 10105 = JCM 12538
Bifidobacterium bifidum NCIMB 41171
Bifidobacterium dentium Bd1
Bifidobacterium pseudocatenulatum DSM 20438 = JCM 1200
Bifidobacterium adolescentis ATCC 15703
Bifidobacterium angulatum DSM 20098 = JCM 7096
Bifidobacterium breve UCC2003
Bifidobacterium longum NCC2705
Bifidobacterium gallicum DSM 20093
Bifidobacterium animalis subsp. lactis BB-12
Cellulomonas flavigena DSM 20109
Cellvibrio gilvus ATCC 13127
Cellulomonas fimi ATCC 484
Isoptericola variabilis 225
Xylanimonas cellulosilytica DSM 15894
Jonesia denitrificans DSM 20603
Sanguibacter keddieii DSM 10542
Acidothermus cellulolyticus 11B
Thermobifida fusca YX
Thermomonospora curvata DSM 43183
Thermobispora bispora DSM 43833
Streptosporangium roseum DSM 43021
Catenulispora acidiphila DSM 44928
Kitasatospora setae KM-6054
Streptomyces cattleya NRRL 8057 = DSM 46488
Streptomyces bingchenggensis BCW-1
Streptomyces himastatinicus ATCC 53653
Streptomyces violaceusniger Tu 4113
Streptomyces sp. C
Streptomyces sp. Mg1
Streptomyces pristinaespiralis ATCC 25486
Streptomyces clavuligerus ATCC 27064
Streptomyces clavuligerus ATCC 27064
Streptomyces clavuligerus ATCC 27064
Streptomyces sp. SirexAA-E
Streptomyces flavogriseus ATCC 33331
Streptomyces griseus subsp. griseus NBRC 13350
Streptomyces albus J1074
Streptomyces sp. SPB78
Streptomyces griseoaurantiacus M045
Streptomyces ghanaensis ATCC 14672
Streptomyces hygroscopicus subsp. jinggangensis 5008
Streptomyces sp. e14
Streptomyces viridochromogenes DSM 40736
Streptomyces griseoflavus Tu4000
Streptomyces coelicolor A3(2)
Streptomyces avermitilis MA-4680
Streptomyces sviceus ATCC 29083
Streptomyces scabiei 87.22
Aeromicrobium marinum DSM 15272
Nocardioides sp. JS614
Nocardioidaceae bacterium Broad-1
Kribbella flavida DSM 17836
Microlunatus phosphovorus NM-1
Propionibacterium freudenreichii subsp. shermanii CIRM-BIA1
Propionibacterium acnes HL037PA2
Propionibacterium acnes SK182B-JCVI
Propionibacterium acnes KPA171202
Frankia sp. EuI1c
Frankia symbiont of Datisca glomerata
Frankia alni ACN14a
Frankia sp. CcI3
Frankia sp. EAN1pec
Nakamurella multipartita DSM 44233
Pseudonocardia dioxanivorans CB1190
Saccharomonospora viridis DSM 43017
Streptomyces sp. AA4
Amycolatopsis mediterranei U32
Saccharopolyspora erythraea NRRL 2338
Actinosynnema mirum DSM 43827
Corynebacterium variabile DSM 44702
Corynebacterium urealyticum DSM 7109
Corynebacterium jeikeium K411
Corynebacterium resistens DSM 45100
Corynebacterium amycolatum SK46
Corynebacterium kroppenstedtii DSM 44385
Corynebacterium efficiens YS-314
Corynebacterium glutamicum ATCC 13032
Corynebacterium glutamicum ATCC 13032
Corynebacterium glucuronolyticum ATCC 51867
Corynebacterium matruchotii ATCC 14266
Corynebacterium diphtheriae PW8
Corynebacterium ulcerans BR-AD22
Corynebacterium pseudotuberculosis I19
Corynebacterium ammoniagenes DSM 20306
Corynebacterium accolens ATCC 49725
Corynebacterium pseudogenitalium ATCC 33035
Corynebacterium aurimucosum ATCC 700975
Corynebacterium striatum ATCC 6940
Corynebacterium lipophiloflavum DSM 44291
Corynebacterium genitalium ATCC 33030
Nocardia cyriacigeorgica GUH-2
Nocardia farcinica IFM 10152
Rhodococcus equi 103S
Rhodococcus erythropolis PR4
Rhodococcus jostii RHA1
Rhodococcus opacus B4
Segniliparus rotundus DSM 44985
Segniliparus rugosus ATCC BAA-974
Mycobacterium abscessus ATCC 19977
Mycobacterium smegmatis str. MC2 155
Mycobacterium rhodesiae NBB3
Mycobacterium sp. JLS
Mycobacterium vanbaalenii PYR-1
Mycobacterium gilvum PYR-GCK
Mycobacterium sp. JDM601
Mycobacterium colombiense CECT 3035
Mycobacterium avium subsp. paratuberculosis K-10
Mycobacterium marinum M
Mycobacterium kansasii ATCC 12478
Mycobacterium leprae TN
Mycobacterium tuberculosis H37Rv
Mycobacterium tuberculosis H37Ra
Mycobacterium tuberculosis H37Ra
Tsukamurella paurometabola DSM 20162
Gordonia neofelifaecis NRRL B-59395
Gordonia alkanivorans NBRC 16433

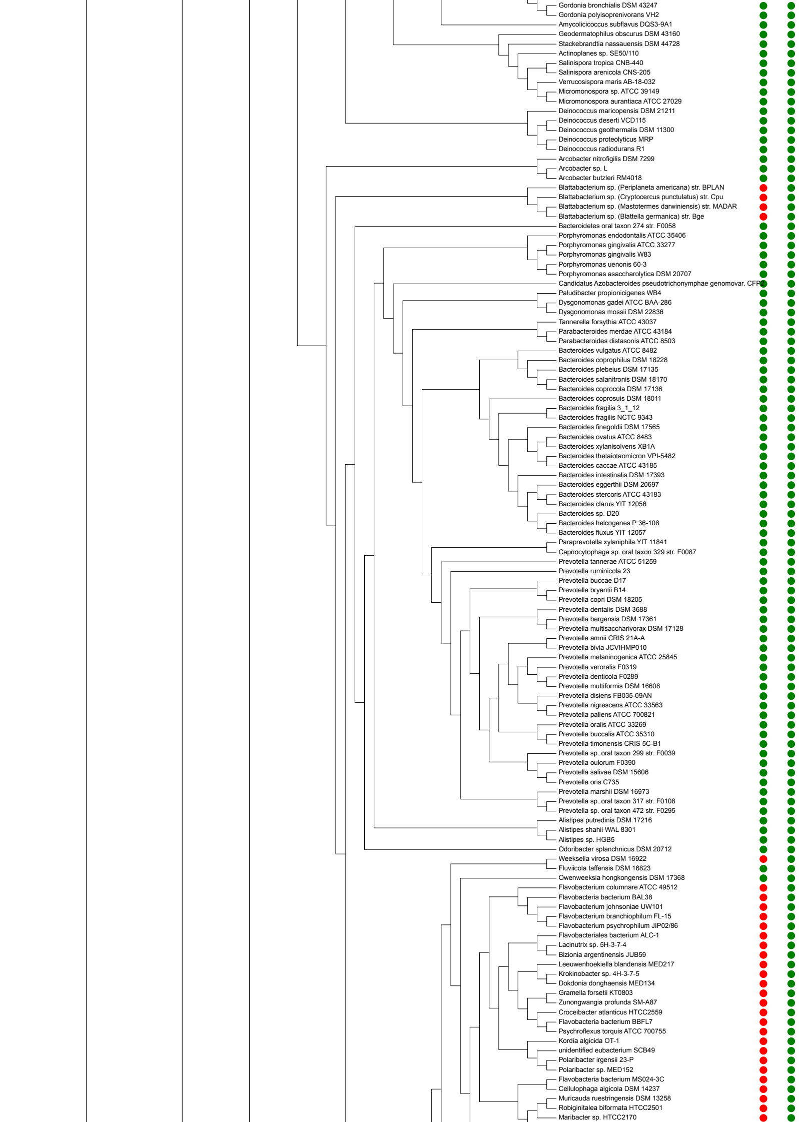
Gordonia bronchialis DSM 43247
Gordonia polyisoprenivorans VH2
Amycolicicoccus subflavus DQS3-9A1
Geodermatophilus obscurus DSM 43160
Stackebrandtia nassauensis DSM 44728
Actinoplanes sp. SE50/110
Salinispora tropica CNB-440
Salinispora arenicola CNS-205
Verrucosispora maris AB-18-032
Micromonospora sp. ATCC 39149
Micromonospora aurantiaca ATCC 27029
Deinococcus maricopensis DSM 21211
Deinococcus deserti VCD115
Deinococcus geothermalis DSM 11300
Deinococcus proteolyticus MRP
Deinococcus radiodurans R1
Arcobacter nitrofigilis DSM 7299
Arcobacter sp. L
Arcobacter butzleri RM4018
Blattabacterium sp. (Periplaneta americana) str. BPLAN
Blattabacterium sp. (Cryptocercus punctulatus) str. Cpu
Blattabacterium sp. (Mastotermes darwiniensis) str. MADAR
Blattabacterium sp. (Blattella germanica) str. Bge
Bacteroidetes oral taxon 274 str. F0058
Porphyromonas endodontalis ATCC 35406
Porphyromonas gingivalis ATCC 33277
Porphyromonas gingivalis W83
Porphyromonas uenonis 60-3
Porphyromonas asaccharolytica DSM 20707
Candidatus Azobacteroides pseudotrichonymphae genomovar. CFP2
Paludibacter propionicigenes WB4
Dysgonomonas gadei ATCC BAA-286
Dysgonomonas mossii DSM 22836
Tannerella forsythia ATCC 43037
Parabacteroides merdae ATCC 43184
Parabacteroides distasonis ATCC 8503
Bacteroides vulgatus ATCC 8482
Bacteroides coprophilus DSM 18228
Bacteroides plebeius DSM 17135
Bacteroides salanitronis DSM 18170
Bacteroides coprocola DSM 17136
Bacteroides coprosuis DSM 18011
Bacteroides fragilis 3_1_12
Bacteroides fragilis NCTC 9343
Bacteroides finegoldii DSM 17565
Bacteroides ovatus ATCC 8483
Bacteroides xylanisolvens XB1A
Bacteroides thetaiotaomicron VPI-5482
Bacteroides caccae ATCC 43185
Bacteroides intestinalis DSM 17393
Bacteroides eggerthii DSM 20697
Bacteroides stercoris ATCC 43183
Bacteroides clarus YIT 12056
Bacteroides sp. D20
Bacteroides helcogenes P 36-108
Bacteroides fluxus YIT 12057
Paraprevotella xylaniphila YIT 11841
Capnocytophaga sp. oral taxon 329 str. F0087
Prevotella tannerae ATCC 51259
Prevotella ruminicola 23
Prevotella buccae D17
Prevotella bryantii B14
Prevotella copri DSM 18205
Prevotella dentalis DSM 3688
Prevotella bergensis DSM 17361
Prevotella multisaccharivorax DSM 17128
Prevotella amnii CRIS 21A-A
Prevotella bivia JCVIHMP010
Prevotella melaninogenica ATCC 25845
Prevotella veroralis F0319
Prevotella denticola F0289
Prevotella multiformis DSM 16608
Prevotella disiens FB035-09AN
Prevotella nigrescens ATCC 33563
Prevotella pallens ATCC 700821
Prevotella oralis ATCC 33269
Prevotella buccalis ATCC 35310
Prevotella timonensis CRIS 5C-B1
Prevotella sp. oral taxon 299 str. F0039
Prevotella oulorum F0390
Prevotella salivae DSM 15606
Prevotella oris C735
Prevotella marshii DSM 16973
Prevotella sp. oral taxon 317 str. F0108
Prevotella sp. oral taxon 472 str. F0295
Alistipes putredinis DSM 17216
Alistipes shahii WAL 8301
Alistipes sp. HGB5
Odoribacter splanchnicus DSM 20712
Weeksella virosa DSM 16922
Fluviicola taffensis DSM 16823
Owenweeksia hongkongensis DSM 17368
Flavobacterium columnare ATCC 49512
Flavobacteria bacterium BAL38
Flavobacterium johnsoniae UW101
Flavobacterium branchiophilum FL-15
Flavobacterium psychrophilum JIP02/86
Flavobacteriales bacterium ALC-1
Lacinutrix sp. 5H-3-7-4
Bizionia argentinensis JUB59
Leeuwenhoekiella blandensis MED217
Krokinobacter sp. 4H-3-7-5
Dokdonia donghaensis MED134
Gramella forsetii KT0803
Zunongwangia profunda SM-A87
Croceibacter atlanticus HTCC2559
Flavobacteria bacterium BBFL7
Psychroflexus torquis ATCC 700755
Kordia algicida OT-1
unidentified eubacterium SCB49
Polaribacter irgensii 23-P
Polaribacter sp. MED152
Flavobacteria bacterium MS024-3C
Cellulophaga algicola DSM 14237
Muricauda ruestringensis DSM 13258
Robiginitalea biformata HTCC2501
Maribacter sp. HTCC2170

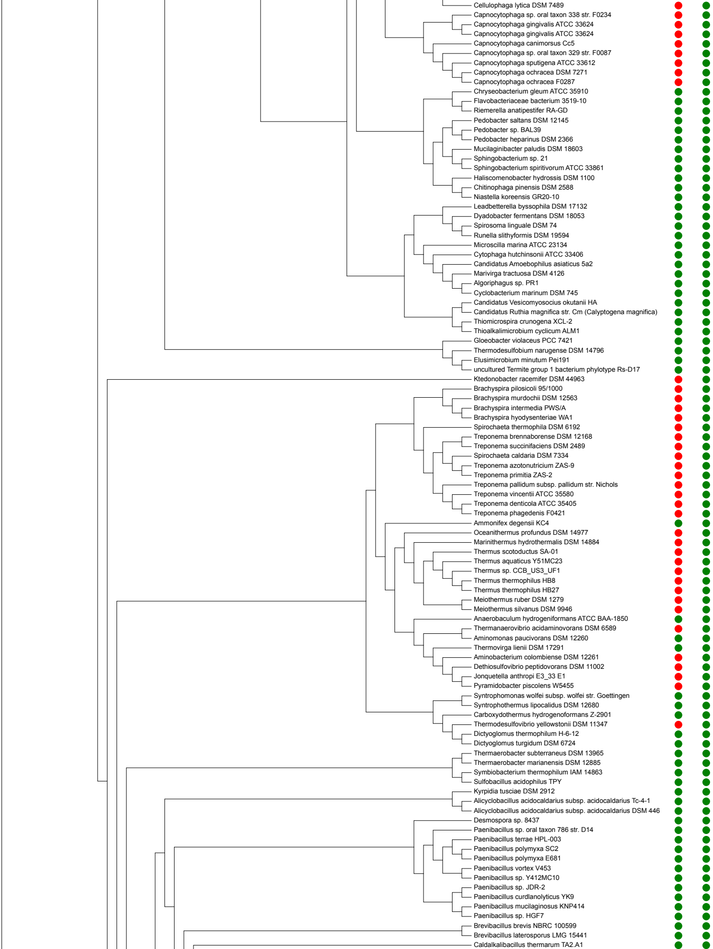
Cellulophaga lytica DSM 7489
Capnocytophaga sp. oral taxon 338 str. F0234
Capnocytophaga gingivalis ATCC 33624
Capnocytophaga gingivalis ATCC 33624
Capnocytophaga canimorsus Cc5
Capnocytophaga sp. oral taxon 329 str. F0087
Capnocytophaga sputigena ATCC 33612
Capnocytophaga ochracea DSM 7271
Capnocytophaga ochracea F0287
Chryseobacterium gleum ATCC 35910
Flavobacteriaceae bacterium 3519-10
Riemerella anatipestifer RA-GD
Pedobacter saltans DSM 12145
Pedobacter sp. BAL39
Pedobacter heparinus DSM 2366
Mucilaginibacter paludis DSM 18603
Sphingobacterium sp. 21
Sphingobacterium spiritivorum ATCC 33861
Haliscomenobacter hydrossis DSM 1100
Chitinophaga pinensis DSM 2588
Niastella koreensis GR20-10
Leadbetterella byssophila DSM 17132
Dyadobacter fermentans DSM 18053
Spirosoma linguale DSM 74
Runella slithyformis DSM 19594
Microscilla marina ATCC 23134
Cytophaga hutchinsonii ATCC 33406
Candidatus Amoebophilus asiaticus 5a2
Marivirga tractuosa DSM 4126
Algoriphagus sp. PR1
Cyclobacterium marinum DSM 745
Candidatus Vesicomyosocius okutanii HA
Candidatus Ruthia magnifica str. Cm (Calyptogena magnifica)
Thiomicrospira crunogena XCL-2
Thioalkalimicrobium cyclicum ALM1
Gloeobacter violaceus PCC 7421
Thermodesulfobium narugense DSM 14796
Elusimicrobium minutum Pei191
uncultured Termite group 1 bacterium phylotype Rs-D17
Ktedonobacter racemifer DSM 44963
Brachyspira pilosicoli 95/1000
Brachyspira murdochii DSM 12563
Brachyspira intermedia PWS/A
Brachyspira hyodysenteriae WA1
Spirochaeta thermophila DSM 6192
Treponema brennaborense DSM 12168
Treponema succinifaciens DSM 2489
Spirochaeta caldaria DSM 7334
Treponema azotonutricium ZAS-9
Treponema primitia ZAS-2
Treponema pallidum subsp. pallidum str. Nichols
Treponema vincentii ATCC 35580
Treponema denticola ATCC 35405
Treponema phagedenis F0421
Ammonifex degensii KC4
Oceanithermus profundus DSM 14977
Marinithermus hydrothermalis DSM 14884
Thermus scotoductus SA-01
Thermus aquaticus Y51MC23
Thermus sp. CCB_US3_UF1
Thermus thermophilus HB8
Thermus thermophilus HB27
Meiothermus ruber DSM 1279
Meiothermus silvanus DSM 9946
Anaerobaculum hydrogeniformans ATCC BAA-1850
Thermanaerovibrio acidaminovorans DSM 6589
Aminomonas paucivorans DSM 12260
Thermovirga lienii DSM 17291
Aminobacterium colombiense DSM 12261
Dethiosulfovibrio peptidovorans DSM 11002
Jonquetella anthropi E3_33 E1
Pyramidobacter piscolens W5455
Syntrophomonas wolfei subsp. wolfei str. Goettingen
Syntrophothermus lipocalidus DSM 12680
Carboxydothermus hydrogenoformans Z-2901
Thermodesulfovibrio yellowstonii DSM 11347
Dictyoglomus thermophilum H-6-12
Dictyoglomus turgidum DSM 6724
Thermaerobacter subterraneus DSM 13965
Thermaerobacter marianensis DSM 12885
Symbiobacterium thermophilum IAM 14863
Sulfobacillus acidophilus TPY
Kyrpidia tusciae DSM 2912
Alicyclobacillus acidocaldarius subsp. acidocaldarius Tc-4-1
Alicyclobacillus acidocaldarius subsp. acidocaldarius DSM 446
Desmospora sp. 8437
Paenibacillus sp. oral taxon 786 str. D14
Paenibacillus terrae HPL-003
Paenibacillus polymyxa SC2
Paenibacillus polymyxa E681
Paenibacillus vortex V453
Paenibacillus sp. Y412MC10
Paenibacillus sp. JDR-2
Paenibacillus curdlanolyticus YK9
Paenibacillus mucilaginosus KNP414
Paenibacillus sp. HGF7
Brevibacillus brevis NBRC 100599
Brevibacillus laterosporus LMG 15441
Caldalkalibacillus thermarum TA2.A1

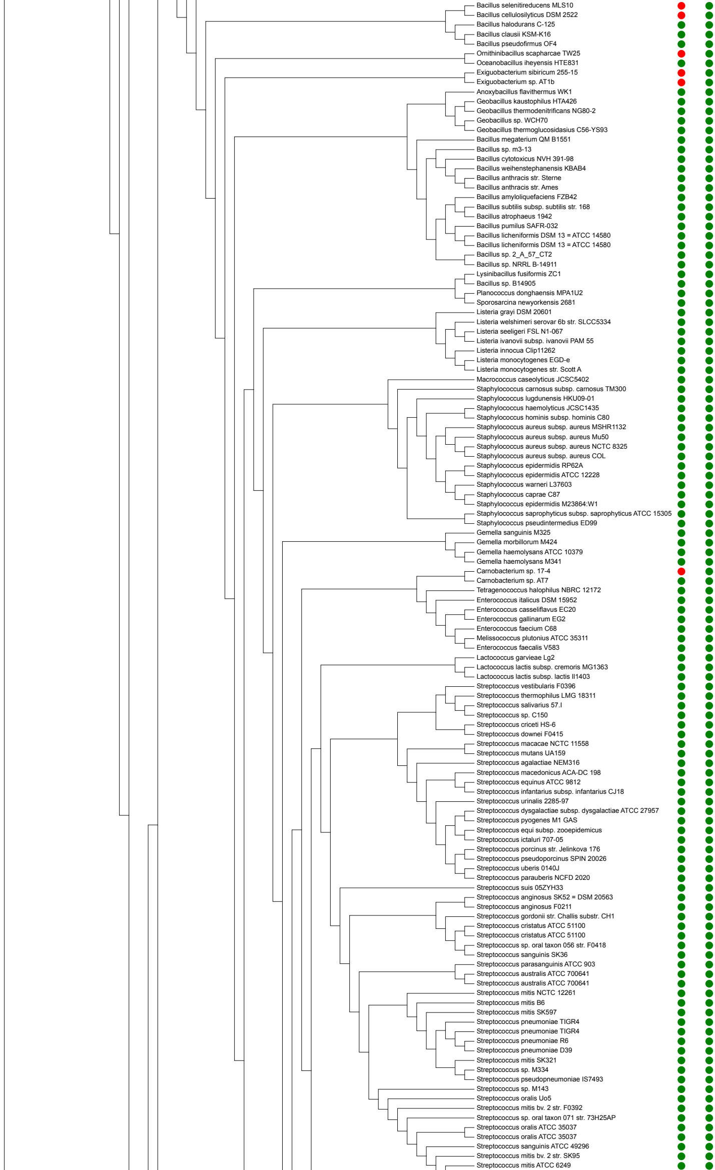
Bacillus selenitireducens MLS10
Bacillus cellulosilyticus DSM 2522
Bacillus halodurans C-125
Bacillus clausii KSM-K16
Bacillus pseudofirmus OF4
Ornithinibacillus scapharcae TW25
Oceanobacillus iheyensis HTE831
Exiguobacterium sibiricum 255-15
Exiguobacterium sp. AT1b
Anoxybacillus flavithermus WK1
Geobacillus kaustophilus HTA426
Geobacillus thermodenitrificans NG80-2
Geobacillus sp. WCH70
Geobacillus thermoglucosidasius C56-YS93
Bacillus megaterium QM B1551
Bacillus sp. m3-13
Bacillus cytotoxicus NVH 391-98
Bacillus weihenstephanensis KBAB4
Bacillus anthracis str. Sterne
Bacillus anthracis str. Ames
Bacillus amyloliquefaciens FZB42
Bacillus subtilis subsp. subtilis str. 168
Bacillus atrophaeus 1942
Bacillus pumilus SAFR-032
Bacillus licheniformis DSM 13 = ATCC 14580
Bacillus licheniformis DSM 13 = ATCC 14580
Bacillus sp. 2_A_57_CT2
Bacillus sp. NRRL B-14911
Lysinibacillus fusiformis ZC1
Bacillus sp. B14905
Planococcus donghaensis MPA1U2
Sporosarcina newyorkensis 2681
Listeria grayi DSM 20601
Listeria welshimeri serovar 6b str. SLCC5334
Listeria seeligeri FSL N1-067
Listeria ivanovii subsp. ivanovii PAM 55
Listeria innocua Clip11262
Listeria monocytogenes EGD-e
Listeria monocytogenes str. Scott A
Macrococcus caseolyticus JCSC5402
Staphylococcus carnosus subsp. carnosus TM300
Staphylococcus lugdunensis HKU09-01
Staphylococcus haemolyticus JCSC1435
Staphylococcus hominis subsp. hominis C80
Staphylococcus aureus subsp. aureus MSHR1132
Staphylococcus aureus subsp. aureus Mu50
Staphylococcus aureus subsp. aureus NCTC 8325
Staphylococcus aureus subsp. aureus COL
Staphylococcus epidermidis RP62A
Staphylococcus epidermidis ATCC 12228
Staphylococcus warneri L37603
Staphylococcus caprae C87
Staphylococcus epidermidis M23864:W1
Staphylococcus saprophyticus subsp. saprophyticus ATCC 15305
Staphylococcus pseudintermedius ED99
Gemella sanguinis M325
Gemella morbillorum M424
Gemella haemolysans ATCC 10379
Gemella haemolysans M341
Carnobacterium sp. 17-4
Carnobacterium sp. AT7
Tetragenococcus halophilus NBRC 12172
Enterococcus italicus DSM 15952
Enterococcus casseliflavus EC20
Enterococcus gallinarum EG2
Enterococcus faecium C68
Melissococcus plutonius ATCC 35311
Enterococcus faecalis V583
Lactococcus garvieae Lg2
Lactococcus lactis subsp. cremoris MG1363
Lactococcus lactis subsp. lactis Il1403
Streptococcus vestibularis F0396
Streptococcus thermophilus LMG 18311
Streptococcus salivarius 57.I
Streptococcus sp. C150
Streptococcus criceti HS-6
Streptococcus downei F0415
Streptococcus macacae NCTC 11558
Streptococcus mutans UA159
Streptococcus agalactiae NEM316
Streptococcus macedonicus ACA-DC 198
Streptococcus equinus ATCC 9812
Streptococcus infantarius subsp. infantarius CJ18
Streptococcus urinalis 2285-97
Streptococcus dysgalactiae subsp. dysgalactiae ATCC 27957
Streptococcus pyogenes M1 GAS
Streptococcus equi subsp. zooepidemicus
Streptococcus ictaluri 707-05
Streptococcus porcinus str. Jelinkova 176
Streptococcus pseudoporcinus SPIN 20026
Streptococcus uberis 0140J
Streptococcus parauberis NCFD 2020
Streptococcus suis 05ZYH33
Streptococcus anginosus SK52 = DSM 20563
Streptococcus anginosus F0211
Streptococcus gordonii str. Challis substr. CH1
Streptococcus cristatus ATCC 51100
Streptococcus cristatus ATCC 51100
Streptococcus sp. oral taxon 056 str. F0418
Streptococcus sanguinis SK36
Streptococcus parasanguinis ATCC 903
Streptococcus australis ATCC 700641
Streptococcus australis ATCC 700641
Streptococcus mitis NCTC 12261
Streptococcus mitis B6
Streptococcus mitis SK597
Streptococcus pneumoniae TIGR4
Streptococcus pneumoniae TIGR4
Streptococcus pneumoniae R6
Streptococcus pneumoniae D39
Streptococcus mitis SK321
Streptococcus sp. M334
Streptococcus pseudopneumoniae IS7493
Streptococcus sp. M143
Streptococcus oralis Uo5
Streptococcus mitis bv. 2 str. F0392
Streptococcus sp. oral taxon 071 str. 73H25AP
Streptococcus oralis ATCC 35037
Streptococcus oralis ATCC 35037
Streptococcus sanguinis ATCC 49296
Streptococcus mitis bv. 2 str. SK95
Streptococcus mitis ATCC 6249

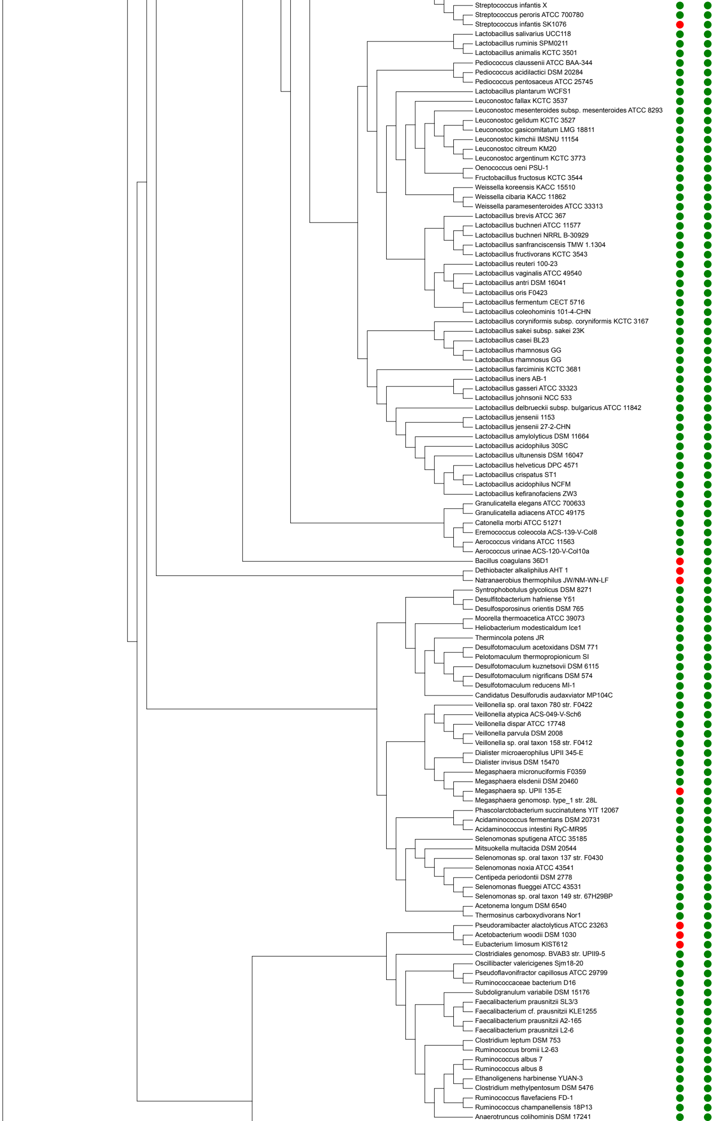
Streptococcus infantis X
Streptococcus peroris ATCC 700780
Streptococcus infantis SK1076
Lactobacillus salivarius UCC118
Lactobacillus ruminis SPM0211
Lactobacillus animalis KCTC 3501
Pediococcus claussenii ATCC BAA-344
Pediococcus acidilactici DSM 20284
Pediococcus pentosaceus ATCC 25745
Lactobacillus plantarum WCFS1
Leuconostoc fallax KCTC 3537
Leuconostoc mesenteroides subsp. mesenteroides ATCC 8293
Leuconostoc gelidum KCTC 3527
Leuconostoc gasicomitatum LMG 18811
Leuconostoc kimchii IMSNU 11154
Leuconostoc citreum KM20
Leuconostoc argentinum KCTC 3773
Oenococcus oeni PSU-1
Fructobacillus fructosus KCTC 3544
Weissella koreensis KACC 15510
Weissella cibaria KACC 11862
Weissella paramesenteroides ATCC 33313
Lactobacillus brevis ATCC 367
Lactobacillus buchneri ATCC 11577
Lactobacillus buchneri NRRL B-30929
Lactobacillus sanfranciscensis TMW 1.1304
Lactobacillus fructivorans KCTC 3543
Lactobacillus reuteri 100-23
Lactobacillus vaginalis ATCC 49540
Lactobacillus antri DSM 16041
Lactobacillus oris F0423
Lactobacillus fermentum CECT 5716
Lactobacillus coleohominis 101-4-CHN
Lactobacillus coryniformis subsp. coryniformis KCTC 3167
Lactobacillus sakei subsp. sakei 23K
Lactobacillus casei BL23
Lactobacillus rhamnosus GG
Lactobacillus rhamnosus GG
Lactobacillus farciminis KCTC 3681
Lactobacillus iners AB-1
Lactobacillus gasseri ATCC 33323
Lactobacillus johnsonii NCC 533
Lactobacillus delbrueckii subsp. bulgaricus ATCC 11842
Lactobacillus jensenii 1153
Lactobacillus jensenii 27-2-CHN
Lactobacillus amylolyticus DSM 11664
Lactobacillus acidophilus 30SC
Lactobacillus ultunensis DSM 16047
Lactobacillus helveticus DPC 4571
Lactobacillus crispatus ST1
Lactobacillus acidophilus NCFM
Lactobacillus kefiranofaciens ZW3
Granulicatella elegans ATCC 700633
Granulicatella adiacens ATCC 49175
Catonella morbi ATCC 51271
Eremococcus coleocola ACS-139-V-Col8
Aerococcus viridans ATCC 11563
Aerococcus urinae ACS-120-V-Col10a
Bacillus coagulans 36D1
Dethiobacter alkaliphilus AHT 1
Natranaerobius thermophilus JW/NM-WN-LF
Syntrophobotulus glycolicus DSM 8271
Desulfitobacterium hafniense Y51
Desulfosporosinus orientis DSM 765
Moorella thermoacetica ATCC 39073
Heliobacterium modesticaldum Ice1
Thermincola potens JR
Desulfotomaculum acetoxidans DSM 771
Pelotomaculum thermopropionicum SI
Desulfotomaculum kuznetsovii DSM 6115
Desulfotomaculum nigrificans DSM 574
Desulfotomaculum reducens MI-1
Candidatus Desulforudis audaxviator MP104C
Veillonella sp. oral taxon 780 str. F0422
Veillonella atypica ACS-049-V-Sch6
Veillonella dispar ATCC 17748
Veillonella parvula DSM 2008
Veillonella sp. oral taxon 158 str. F0412
Dialister microaerophilus UPII 345-E
Dialister invisus DSM 15470
Megasphaera micronuciformis F0359
Megasphaera elsdenii DSM 20460
Megasphaera sp. UPII 135-E
Megasphaera genomosp. type_1 str. 28L
Phascolarctobacterium succinatutens YIT 12067
Acidaminococcus fermentans DSM 20731
Acidaminococcus intestini RyC-MR95
Selenomonas sputigena ATCC 35185
Mitsuokella multacida DSM 20544
Selenomonas sp. oral taxon 137 str. F0430
Selenomonas noxia ATCC 43541
Centipeda periodontii DSM 2778
Selenomonas flueggei ATCC 43531
Selenomonas sp. oral taxon 149 str. 67H29BP
Acetonema longum DSM 6540
Thermosinus carboxydivorans Nor1
Pseudoramibacter alactolyticus ATCC 23263
Acetobacterium woodii DSM 1030
Eubacterium limosum KIST612
Clostridiales genomosp. BVAB3 str. UPII9-5
Oscillibacter valericigenes Sjm18-20
Pseudoflavonifractor capillosus ATCC 29799
Ruminococcaceae bacterium D16
Subdoligranulum variabile DSM 15176
Faecalibacterium prausnitzii SL3/3
Faecalibacterium cf. prausnitzii KLE1255
Faecalibacterium prausnitzii A2-165
Faecalibacterium prausnitzii L2-6
Clostridium leptum DSM 753
Ruminococcus bromii L2-63
Ruminococcus albus 7
Ruminococcus albus 8
Ethanoligenens harbinense YUAN-3
Clostridium methylpentosum DSM 5476
Ruminococcus flavefaciens FD-1
Ruminococcus champanellensis 18P13
Anaerotruncus colihominis DSM 17241

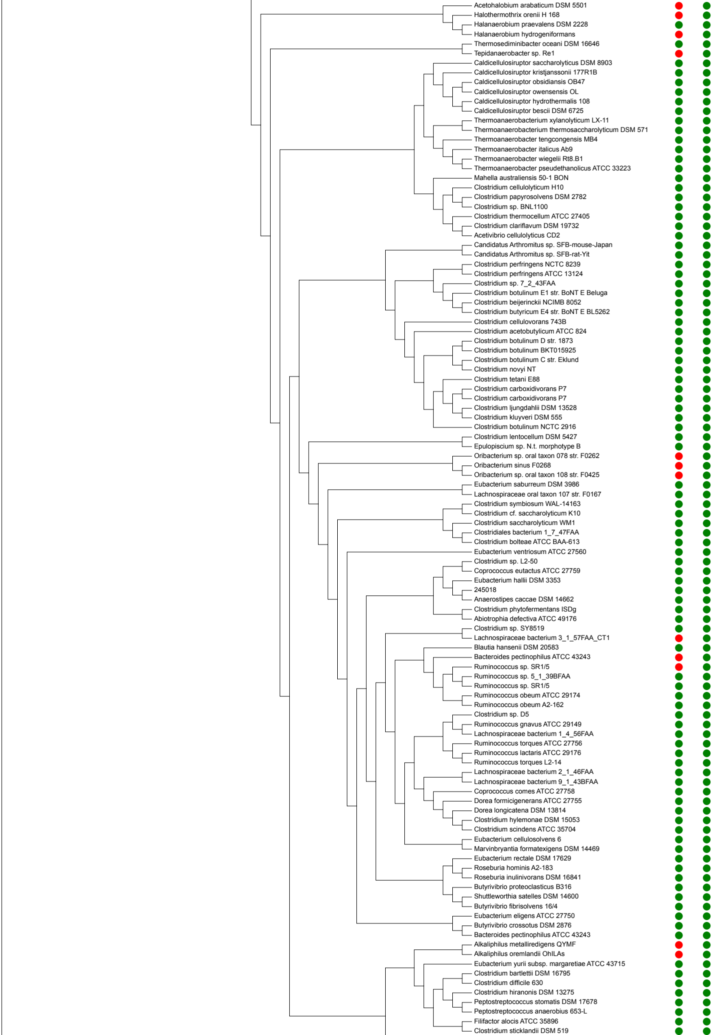
Acetohalobium arabaticum DSM 5501
Halothermothrix orenii H 168
Halanaerobium praevalens DSM 2228
Halanaerobium hydrogeniformans
Thermosediminibacter oceani DSM 16646
Tepidanaerobacter sp. Re1
Caldicellulosiruptor saccharolyticus DSM 8903
Caldicellulosiruptor kristjanssonii 177R1B
Caldicellulosiruptor obsidiansis OB47
Caldicellulosiruptor owensensis OL
Caldicellulosiruptor hydrothermalis 108
Caldicellulosiruptor bescii DSM 6725
Thermoanaerobacterium xylanolyticum LX-11
Thermoanaerobacterium thermosaccharolyticum DSM 571
Thermoanaerobacter tengcongensis MB4
Thermoanaerobacter italicus Ab9
Thermoanaerobacter wiegelii Rt8.B1
Thermoanaerobacter pseudethanolicus ATCC 33223
Mahella australiensis 50-1 BON
Clostridium cellulolyticum H10
Clostridium papyrosolvens DSM 2782
Clostridium sp. BNL1100
Clostridium thermocellum ATCC 27405
Clostridium clariflavum DSM 19732
Acetivibrio cellulolyticus CD2
Candidatus Arthromitus sp. SFB-mouse-Japan
Candidatus Arthromitus sp. SFB-rat-Yit
Clostridium perfringens NCTC 8239
Clostridium perfringens ATCC 13124
Clostridium sp. 7_2_43FAA
Clostridium botulinum E1 str. BoNT E Beluga
Clostridium beijerinckii NCIMB 8052
Clostridium butyricum E4 str. BoNT E BL5262
Clostridium cellulovorans 743B
Clostridium acetobutylicum ATCC 824
Clostridium botulinum D str. 1873
Clostridium botulinum BKT015925
Clostridium botulinum C str. Eklund
Clostridium novyi NT
Clostridium tetani E88
Clostridium carboxidivorans P7
Clostridium carboxidivorans P7
Clostridium ljungdahlii DSM 13528
Clostridium kluyveri DSM 555
Clostridium botulinum NCTC 2916
Clostridium lentocellum DSM 5427
Epulopiscium sp. N.t. morphotype B
Oribacterium sp. oral taxon 078 str. F0262
Oribacterium sinus F0268
Oribacterium sp. oral taxon 108 str. F0425
Eubacterium saburreum DSM 3986
Lachnospiraceae oral taxon 107 str. F0167
Clostridium symbiosum WAL-14163
Clostridium cf. saccharolyticum K10
Clostridium saccharolyticum WM1
Clostridiales bacterium 1_7_47FAA
Clostridium bolteae ATCC BAA-613
Eubacterium ventriosum ATCC 27560
Clostridium sp. L2-50
Coprococcus eutactus ATCC 27759
Eubacterium hallii DSM 3353
245018
Anaerostipes caccae DSM 14662
Clostridium phytofermentans ISDg
Abiotrophia defectiva ATCC 49176
Clostridium sp. SY8519
Lachnospiraceae bacterium 3_1_57FAA_CT1
Blautia hansenii DSM 20583
Bacteroides pectinophilus ATCC 43243
Ruminococcus sp. SR1/5
Ruminococcus sp. 5_1_39BFAA
Ruminococcus sp. SR1/5
Ruminococcus obeum ATCC 29174
Ruminococcus obeum A2-162
Clostridium sp. D5
Ruminococcus gnavus ATCC 29149
Lachnospiraceae bacterium 1_4_56FAA
Ruminococcus torques ATCC 27756
Ruminococcus lactaris ATCC 29176
Ruminococcus torques L2-14
Lachnospiraceae bacterium 2_1_46FAA
Lachnospiraceae bacterium 9_1_43BFAA
Coprococcus comes ATCC 27758
Dorea formicigenerans ATCC 27755
Dorea longicatena DSM 13814
Clostridium hylemonae DSM 15053
Clostridium scindens ATCC 35704
Eubacterium cellulosolvens 6
Marvinbryantia formatexigens DSM 14469
Eubacterium rectale DSM 17629
Roseburia hominis A2-183
Roseburia inulinivorans DSM 16841
Butyrivibrio proteoclasticus B316
Shuttleworthia satelles DSM 14600
Butyrivibrio fibrisolvens 16/4
Eubacterium eligens ATCC 27750
Butyrivibrio crossotus DSM 2876
Bacteroides pectinophilus ATCC 43243
Alkaliphilus metalliredigens QYMF
Alkaliphilus oremlandii OhILAs
Eubacterium yurii subsp. margaretiae ATCC 43715
Clostridium bartlettii DSM 16795
Clostridium difficile 630
Clostridium hiranonis DSM 13275
Peptostreptococcus stomatis DSM 17678
Peptostreptococcus anaerobius 653-L
Filifactor alocis ATCC 35896
Clostridium sticklandii DSM 519

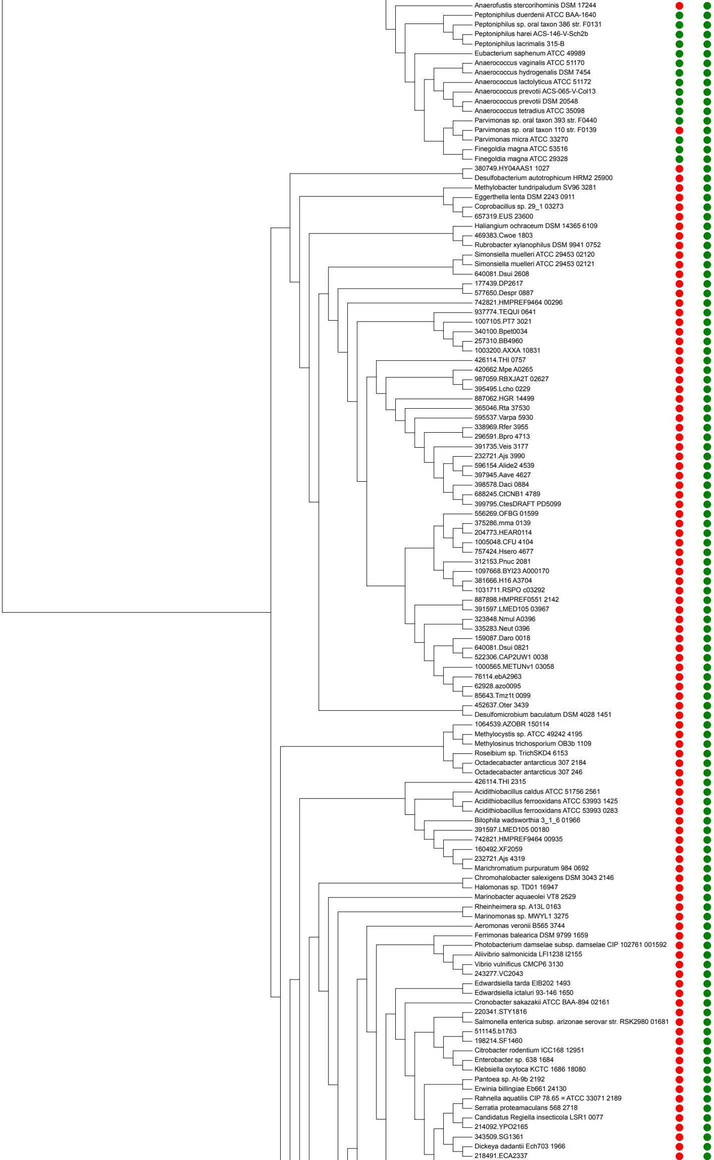

Anaerofustis stercorihominis DSM 17244
Peptoniphilus duerdenii ATCC BAA-1640
Peptoniphilus sp. oral taxon 386 str. F0131
Peptoniphilus harei ACS-146-V-Sch2b
Peptoniphilus lacrimalis 315-B
Eubacterium saphenum ATCC 49989
Anaerococcus vaginalis ATCC 51170
Anaerococcus hydrogenalis DSM 7454
Anaerococcus lactolyticus ATCC 51172
Anaerococcus prevotii ACS-065-V-Col13
Anaerococcus prevotii DSM 20548
Anaerococcus tetradius ATCC 35098
Parvimonas sp. oral taxon 393 str. F0440
Parvimonas sp. oral taxon 110 str. F0139
Parvimonas micra ATCC 33270
Finegoldia magna ATCC 53516
Finegoldia magna ATCC 29328
380749.HY04AAS1 1027
Desulfobacterium autotrophicum HRM2 25900
Methylobacter tundripaludum SV96 3281
Eggerthella lenta DSM 2243 0911
Coprobacillus sp. 29_1 03273
657319.EUS 23600
Haliangium ochraceum DSM 14365 6109
469383.Cwoe 1803
Rubrobacter xylanophilus DSM 9941 0752
Simonsiella muelleri ATCC 29453 02120
Simonsiella muelleri ATCC 29453 02121
640081.Dsui 2608
177439.DP2617
577650.Despr 0887
742821.HMPREF9464 00296
937774.TEQUI 0641
1007105.PT7 3021
340100.Bpet0034
257310.BB4960
1003200.AXXA 10831
426114.THI 0757
420662.Mpe A0265
987059.RBXJA2T 02627
395495.Lcho 0229
887062.HGR 14499
365046.Rta 37530
595537.Varpa 5930
338969.Rfer 3955
296591.Bpro 4713
391735.Veis 3177
232721.Ajs 3990
596154.Alide2 4539
397945.Aave 4627
398578.Daci 0884
688245.CtCNB1 4789
399795.CtesDRAFT PD5099
556269.OFBG 01599
375286.mma 0139
204773.HEAR0114
1005048.CFU 4104
757424.Hsero 4677
312153.Pnuc 2081
1097668.BYI23 A000170
381666.H16 A3704
1031711.RSPO c03292
887898.HMPREF0551 2142
391597.LMED105 03967
323848.Nmul A0396
335283.Neut 0396
159087.Daro 0018
640081.Dsui 0821
522306.CAP2UW1 0038
1000565.METUNv1 03058
76114.ebA2963
62928.azo0095
85643.Tmz1t 0099
452637.Oter 3439
Desulfomicrobium baculatum DSM 4028 1451
1064539.AZOBR 150114
Methylocystis sp. ATCC 49242 4195
Methylosinus trichosporium OB3b 1109
Roseibium sp. TrichSKD4 6153
Octadecabacter antarcticus 307 2184
Octadecabacter antarcticus 307 246
426114.THI 2315
Acidithiobacillus caldus ATCC 51756 2561
Acidithiobacillus ferrooxidans ATCC 53993 1425
Acidithiobacillus ferrooxidans ATCC 53993 0283
Bilophila wadsworthia 3_1_6 01966
391597.LMED105 00180
742821.HMPREF9464 00935
160492.XF2059
232721.Ajs 4319
Marichromatium purpuratum 984 0692
Chromohalobacter salexigens DSM 3043 2146
Halomonas sp. TD01 16947
Marinobacter aquaeolei VT8 2529
Rheinheimera sp. A13L 0163
Marinomonas sp. MWYL1 3275
Aeromonas veronii B565 3744
Ferrimonas balearica DSM 9799 1659
Photobacterium damselae subsp. damselae CIP 102761 001592
Aliivibrio salmonicida LFI1238 I2155
Vibrio vulnificus CMCP6 3130
243277.VC2043
Edwardsiella tarda EIB202 1493
Edwardsiella ictaluri 93-146 1650
Cronobacter sakazakii ATCC BAA-894 02161
220341.STY1816
Salmonella enterica subsp. arizonae serovar str. RSK2980 01681
511145.b1763
198214.SF1460
Citrobacter rodentium ICC168 12951
Enterobacter sp. 638 1684
Klebsiella oxytoca KCTC 1686 18080
Pantoea sp. At-9b 2192
Erwinia billingiae Eb661 24130
Rahnella aquatilis CIP 78.65 = ATCC 33071 2189
Serratia proteamaculans 568 2718
Candidatus Regiella insecticola LSR1 0077
214092.YPO2165
343509.SG1361
Dickeya dadantii Ech703 1966
218491.ECA2337

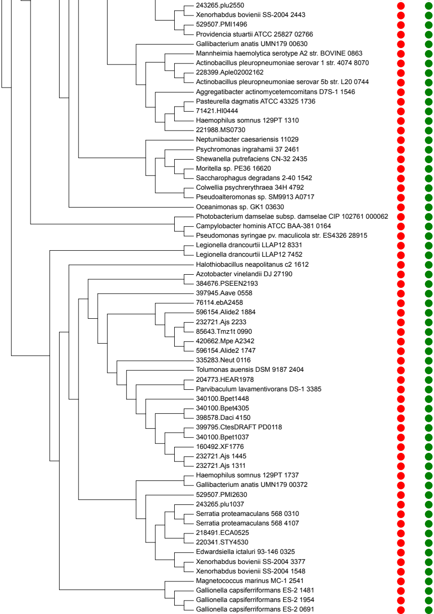
243265.plu2550
Xenorhabdus bovienii SS-2004 2443
529507.PMI1496
Providencia stuartii ATCC 25827 02766
Gallibacterium anatis UMN179 00630
Mannheimia haemolytica serotype A2 str. BOVINE 0863
Actinobacillus pleuropneumoniae serovar 1 str. 4074 8070
228399.Aple02002162
Actinobacillus pleuropneumoniae serovar 5b str. L20 0744
Aggregatibacter actinomycetemcomitans D7S-1 1546
Pasteurella dagmatis ATCC 43325 1736
71421.HI0444
Haemophilus somnus 129PT 1310
221988.MS0730
Neptuniibacter caesariensis 11029
Psychromonas ingrahamii 37 2461
Shewanella putrefaciens CN-32 2435
Moritella sp. PE36 16620
Saccharophagus degradans 2-40 1542
Colwellia psychrerythraea 34H 4792
Pseudoalteromonas sp. SM9913 A0717
Oceanimonas sp. GK1 03630
Photobacterium damselae subsp. damselae CIP 102761 000062
Campylobacter hominis ATCC BAA-381 0164
Pseudomonas syringae pv. maculicola str. ES4326 28915
Legionella drancourtii LLAP12 8331
Legionella drancourtii LLAP12 7452
Halothiobacillus neapolitanus c2 1612
Azotobacter vinelandii DJ 27190
384676.PSEEN2193
397945.Aave 0558
76114.ebA2458
596154.Alide2 1884
232721.Ajs 2233
85643.Tmz1t 0990
420662.Mpe A2342
596154.Alide2 1747
335283.Neut 0116
Tolumonas auensis DSM 9187 2404
204773.HEAR1978
Parvibaculum lavamentivorans DS-1 3385
340100.Bpet1448
340100.Bpet4305
398578.Daci 4150
399795.CtesDRAFT PD0118
340100.Bpet1037
160492.XF1776
232721.Ajs 1445
232721.Ajs 1311
Haemophilus somnus 129PT 1737
Gallibacterium anatis UMN179 00372
529507.PMI2630
243265.plu1037
Serratia proteamaculans 568 0310
Serratia proteamaculans 568 4107
218491.ECA0525
220341.STY4530
Edwardsiella ictaluri 93-146 0325
Xenorhabdus bovienii SS-2004 3377
Xenorhabdus bovienii SS-2004 1548
Magnetococcus marinus MC-1 2541
Gallionella capsiferriformans ES-2 1481
Gallionella capsiferriformans ES-2 1954
Gallionella capsiferriformans ES-2 0691
